# Dissolved Black Carbon Facilitates the Photodegradation of Microplastics via Molecular Weight-Dependent Generation of Reactive Intermediates

**DOI:** 10.1021/acs.est.4c03831

**Published:** 2024-08-12

**Authors:** Qin Ou, Yanghui Xu, Xintu Wang, Jan Peter van der Hoek, Guo Yu, Gang Liu

**Affiliations:** † Key Laboratory of Drinking Water Science and Technology, Research Centre for Eco-Environmental Sciences, Chinese Academy of Sciences, Beijing 100085, PR China; ‡ Section of Sanitary Engineering, Department of Water Management, Faculty of Civil Engineering and Geosciences, Delft University of Technology, Delft, CN 2628, The Netherlands; § College of Environmental Science and Engineering, Guilin University of Technology, Guangxi 541004, China; ∥ Department Research & Innovation Waternet, P.O. Box 94370 GJ Amsterdam 1090, The Netherlands; ⊥ University of Chinese Academy of Sciences, Beijing 101408, China

**Keywords:** microplastics, photodegradation, dissolved black carbon, molecular weight, reactive intermediates

## Abstract

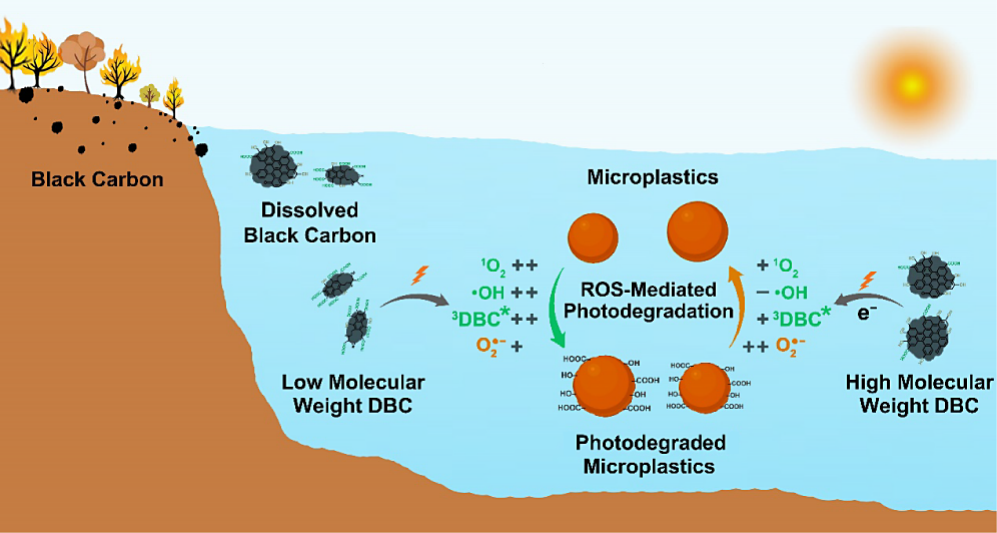

Photodegradation of microplastics (MPs) induced by sunlight plays a crucial role in determining their transport, fate, and impacts in aquatic environments. Dissolved black carbon (DBC), originating from pyrolyzed carbon, can potentially mediate the photodegradation of MPs owing to its potent photosensitization capacity. This study examined the impact of pyrolyzed wood derived DBC (5 mg C/L) on the photodegradation of polystyrene (PS) MPs in aquatic solutions under UV radiation. It revealed that the photodegradation of PS MPs primarily occurred at the benzene ring rather than the aliphatic segments due to the fast attack of hydroxyl radical (•OH) and singlet oxygen (^1^O_2_) on the benzene ring. The photosensitivity of DBC accelerated the degradation of PS MPs, primarily attributed to the increased production of •OH, ^1^O_2_, and triplet-excited state DBC (^3^DBC*). Notably, DBC-mediated photodegradation was related to its molecular weight (MW) and chemical properties. Low MW DBC (<3 kDa) containing more carbonyl groups generated more •OH and ^1^O_2_, accelerating the photodegradation of MPs. Nevertheless, higher aromatic phenols in high MW DBC (>30 kDa) scavenged •OH and generated more O_2_•^–^, inhibiting the photodegradation of MPs. Overall, this study offered valuable insights into UV-induced photodegradation of MPs and highlighted potential impacts of DBC on the transformation of MPs.

## Abstract 2

The investigation into the effect of DBC on the photodegradation of MPs contributes to our understanding of the photodegradation mechanism of MPs and has implications for comprehending their fate under natural disturbances.

## 1 Introduction

The increasing frequency and severity of wildfires, closely linked to climate change, are expected to intensify as climate change continues.^1,2^ The incomplete combustion of vegetation and soil organic material during wildfires produces black carbon (BC).^3,4^ It is estimated that the burning of biomass predominantly contributes to the global production of BC, generating approximately 114–383 Tg per year.^5^ Dissolved black carbon (DBC), defined as the water-soluble fraction of pyrolyzed carbon or black carbon (BC), is a significant component of natural dissolved organic matter (DOM).^6,7^ DBC represents approximately 10% of the global riverine dissolved organic carbon (DOC) flux to oceans,^7^ highlighting the potential significance of DBC in environmental contexts.

DBC exhibits distinct chemical and structural characteristics due to its unique origins and processes of formation, which differ considerably from those of terrestrial and autochthonous DOMs.^8,9^ It primarily consists of fused aromatic rings and hydrophilic oxygen-based functional groups, such as carboxylic and phenolic.^10,11^ This structure confers DBC with notable photoreactivity, capable of generating various reactive species (RIs),^11,12^ such as •OH, superoxide (O_2_•^–^), singlet oxygen (^1^O_2_), and triplet state DBC (^3^DBC*).^11−14^ The photosensitivity of DBC has been extensively studied for its significant role in the removal of organic contaminants in environments, such as 17β-estradiol, Hg (II) and imidacloprid.^8,14,15^

As emerging contaminants, microplastics (MPs) (<5 mm in size) has raised alarms due to their ubiquitous presence and the potential environmental hazards they pose.^16−200^ Long-term exposure to environmental conditions can lead to further degradation or breakdown of MPs through light, heat, and biological process.^19,20^ Photodegradation is widely recognized as the predominant degradation process for MPs, leading to reduced particle size, alterations in surface features, and the leaching of chemicals.^21,22^ These alterations could further influence the transport, fate, and toxicity of MPs in environments.^20^ Due to its environmental significance, the photodegradation process of MPs has garnered significant attention in recent years.

MPs do not undergo photodegradation in environments alone; their degradation often involves the participation of natural compounds, such as ions (Cl^–^, NO_3_^–^),^23−25^ minerals (kaolinite, montmorillonite, and iron (hydr)oxides),^26−28^ and DOM, such as humic acid (HA) and fulvic acid (FA).^23,29,30^ For instance, HA and FA have been reported to inhibit the photodegradation of polypropylene MPs by acting as optical light filters.^30^ However, the presence of HA and FA has been observed to accelerate the aging process of polystyrene (PS) MPs, with FA exhibiting a more pronounced effect than HA.^31^ Compared to well-studied natural DOMs, DBC shows higher aromatic structure content and smaller molecular sizes.^8,32^ As reported, DBC shows a notably higher yield of ^3^DBC* and ^1^O_2_ compared to natural DOMs.^9,32^ Despite this, knowledge regrading the impact of DBC on the photodegradation of MPs in water is scarce, leaving uncertainties about whether and how DBC affects the photochemical transformation of MPs.

This study aimed to investigate the influence of DBC on the photodegradation of MPs. Artificial accelerated photoaging experiments were conducted to simulate the photodegradation of PS MPs in the absence and presence of DBC. The extent of photodegradation and its effects were assessed and compared using scanning electron microscopy (SEM) and Fourier-transform infrared spectroscopy (FTIR). Subsequently, DBC was fractionated based on molecular weights (MWs) to investigate how specific components of DBC influence the photodegradation process. The chemical probe methods and RI scavenger experiments were conducted to determine the key RIs involved. Furthermore, the photodegradation mechanism of PS MPs and the mediating mechanism of DBC was revealed by combining two-dimensional correlation spectroscopy (2D-COS) analysis.

## 2 Materials and Methods

### 2.1 Chemicals and Materials

The PS MP powder, within a 50–450 μm size range, was obtained from Dongguan Zhangmutou Plastic Industry Development Co., Ltd., China. These MPs were subsequently cleaned with ethanol and deionized water three times to remove possible organic impurities, and then dried at 40 °C for 48 h and stored under dry, dark condition before usage. Pyrolyzed carbon derived from wood was sourced from Nanjing Zhironglian Co., Ltd. Filtration membranes of 0.45 and 0.22 μm PES were acquired from Tianjin Jinteng Experimental Equipment Co., Ltd., China, along with 30 kDa and 3 kDa ultrafiltration membranes from RisingSun Membrane Technology (Beijing) Co., Ltd., China. Superoxide dismutase (SOD) from bovine erythrocytes (5000 units/mg), isopropyl alcohol (IPA, AR), furfuryl alcohol (FFA, 98%), 2,3-bis(2-methoxy-4-nitro-5-sulfophenyl)-2H-tetrazolium-5-carboxanilide (XTT, > 90%), 2,4,6-trimethylphenol (TMP, 97%), NaH_2_PO_4_, and NaOH, were supplied by Macklin Biochemical Co., Ltd., China. All solutions were formulated with ultrapure water (18.2 MΩ cm^–1^, Milli-Q, Millipore).

### 2.2 DBC Preparation

DBC was derived from wood-derived pyrolyzed carbon (Nanjing Zhironglian Co., Ltd.), produced by pyrolyzing wood at 500 °C in a muffle furnace under limited oxygen for 2 h with a single run of 3 kg. The raw material of the wood is tree trunk and branch of deciduous plants, which were chosen because it closely resembles environmental DBC produced by natural wildfires.^33,34^ The pyrolyzed carbon was then pulverized into a fine powder using a high-speed blender. 60 g of this powder were mixed with 500 mL of Milli-Q water in a glass bottle and ultrasonicated for 30 min. During this process, the bottle was occasionally shaken to enhance extract efficiency, yielding the pyrolyzed carbon extract. Bulk DBC was collected as a filtrate after passing this extract through a 0.45 μm PES filter membrane (prewashed with 500 mL of ultrapure water to eliminate any organic residues from the membrane). Bulk DBC were isolated via two successive ultrafiltration steps using 30 kDa and 3 kDa membranes (each prerinsed with 500 mL of Milli-Q water) to gain DBC fractions (<3 kDa, 3–30 kDa, 30 kDa-0.45 μm, Figure S1). The characteristics of bulk DBC and DBC fractions were analyzed using ultraviolet–visible (UV–vis) absorption spectroscopy (UH4150, Hitachi), total organic carbon analyzer (TOC-L, Shimadzu), and fluorescence spectroscopy (F-7000, Hitachi).

### 2.3 Photochemical Experiments

Photochemical experiments designed to accelerate the photoaging of MPs were conducted in a UV chamber, which was maintained at approximately 30 °C by circulating cooling water. The chamber was equipped with a 1000 W mercury lamp (Figure S2) (Dongguan Ergu photoelectric Technology Co., LTD, China). It was determined that 3 h of the irradiation on PS MPs was equivalent to ∼3 weeks of outdoor natural weathering on a rooftop during Beijing’s summer, based on the comparison of the aging-related carbonyl index (CI) value (Text S5).^30,35^ Additionally, the chamber housed several 100 mL quartz tubes and magnetic stirrers. Each quartz tube, containing 0.25 g of PS MPs in 100 mL of a pH 7.0 buffer solution (2.5 mM KH_2_PO_4_, pH adjusted with 1 M NaOH), was subjected to 10 min of ultrasonication. This study aimed to characterize the photodegradation properties of PS MPs, so the tested MP amounts were intentionally higher than those found in natural water.^36,37^ PS MPs were then exposed to UV light while being agitated at 200 rpm, both in the absence (PS group) and presence of bulk DBC (5 mg C/L, bulk + PS group). 200 rpm was used to ensure uniform exposure of MPs to UV light and prevent sedimentation. This speed range was commonly used in similar studies, typically ranging from 150 to 250 rpm in the literature.^25,31,37^ The preliminary results also indicated that the agitation within 24 h did not lead to the surface oxidation of PS MPs. The PS MPs samples were collected at specified intervals (0, 3, 6, 12, 18, 24, 36, and 48 h) by filtering the suspension through a 1 μm stainless steel filter membrane after sacrificing the reacted solutions. The filtered MPs were washed three times with ethanol and ultrapure water to eliminate the possibility that the observed effects of DBC on the PS MPs were due to physical adsorption.^36^ Subsequently, they were dried at 40 °C for 48 h and stored in the dark until further analysis. Control experiments, excluding UV irradiation and labeled as PS-dark, were carried out in parallel by covering the tubes with aluminum foil. Photodegradation of fluorescent substances in DBC fractions with varying MWs (<3 kDa, 3–30 kDa, 30 kDa-0.45 μm, and bulk) showed <3 kDa degraded most rapidly, followed by 3–30 kDa and then 30 kDa-0.45 μm, disappearing within 24 h (Figure S3). Further investigation into the effect of different DBC fractions on the photodegradation of MPs was conducted within a 24 h time frame. All treatments were replicated three times.

### 2.4 Characterization of MPs and DBC

The surface morphology of the MPs was investigated using SEM (Quattro, FEI). The size distribution of the particles was determined by analyzing SEM images with ImageJ software (version 1.2.5, Fudan University), 90 particles were randomly selected for this analysis (Figure S4),^30^ and laser particle size analyzer (LPSA; Mastersizer 3000, Malvern). Surface functional groups were explored through FTIR spectroscopy (Nicolet iN10MX, Thermo Fisher Scientific). Automatic baseline correction was performed after FTIR spectra data acquisition by software OMNIC 8.2. The 2D-COS analysis was conducted to gain a deeper understanding of the changes in the functional groups of the MPs. Detailed information regarding 2D-COS analysis can be referenced elsewhere.^26,31,38^ DOC of DBC and the PS leaching solution was quantified using a TOC analyzer. Additionally, changes in the fluorescent properties of DBC and the PS leaching solution during irradiation were assessed using excitation–emission matrices (EEMs).

### 2.5 RI Identification

To delve into the photodegradation mechanisms of PS MPs mediated by DBC, quenching tests were performed by introducing specific RI scavengers into the aging suspension. IPA (10 mM), SOD (3 mg/L), FFA (10 mM), and TMP (0.1 mM) were used to quench •OH, O_2_•^–^, ^1^O_2_, and ^3^DBC*/^3^PS*, respectively. Each scavenger was added separately to the suspension. The quenching experiments lasted for 24 h, with the scavengers being replenished every 12 h to counteract their depletion. Following the 24 h period, the bottles were processed to collect the PS MPs for subsequent analysis.

The concentrations of RIs (•OH, O_2_^•–^, ^1^O_2_, and ^3^DBC*/^3^PS*) in the suspension were measured through chemical probe techniques, utilizing NB, XTT, FFA and TMP as probe, respectively. Likewise, a 100 mL suspension of 2.5 g/L PS MPs, spiked with distinct probe molecule (0.2 mM NB, 0.05 mM XTT, 0.5 mM FFA, and 0.22 mM TMP),^8,30,39^ was subjected to irradiation under consistent conditions. Controls without irradiation, labeled as PS MPs-dark or bulk DBC-dark, were also included in the study. 1 mL of mixture was extracted at predetermined intervals and filtered through a 0.22 μm PES membrane for subsequent analyses. The concentrations of FFA, TMP, and NB were determined using high-performance liquid chromatography (HPLC, LC-20A, Shimadzu). Changes in XTT concentrations were monitored with a UV–vis spectrometer. The details for the RI measurements are shown in the Texts S1 and S2.

### 2.6 Statistic Analysis

Student’s *t* test and one-way analysis of variance (ANOVA) followed by Tukey HSD test were performed to assess difference using origin software (version 2022) and SPSS (IBM, USA). Results with *P*-values <0.05 are reported as significant. Data are expressed as mean values ± standard deviation (SD).

## 3 Results and Discussion

### 3.1 PS MP Photodegradation Under UV Radiation

Figure S5 presents digital images of PS MPs capturing their state before and after photoaging. In all photoaged samples, noticeable yellow color was observed, with the intensity increasing as the irradiation time progressed. This observation indicated that photodegradation stimulated the formation of chromophores. Complementary SEM images (Figure S6) depicted the surface of pristine PS MPs as seemingly rough and irregular, exhibiting multiple cracks resulting from the mechanical grinding in the manufacturing process.^23,40^ Notably, their surface tended to smooth out after photoaging, likely due to surface stripping in the photodegradation process.^40^ After 48 h of photodegradation, the average particle size of PS MPs decreased from 281 to 233 μm (Table S1), indicating surface erosion of PS MPs due to the photodegradation process. Concurrently, leaching of molecular fragments, specifically low MW degradation products, into the water was observed, as demonstrated by an increase in DOC and fluorescence intensity, as shown in Figures S7 and S8.

**Figure 1:**
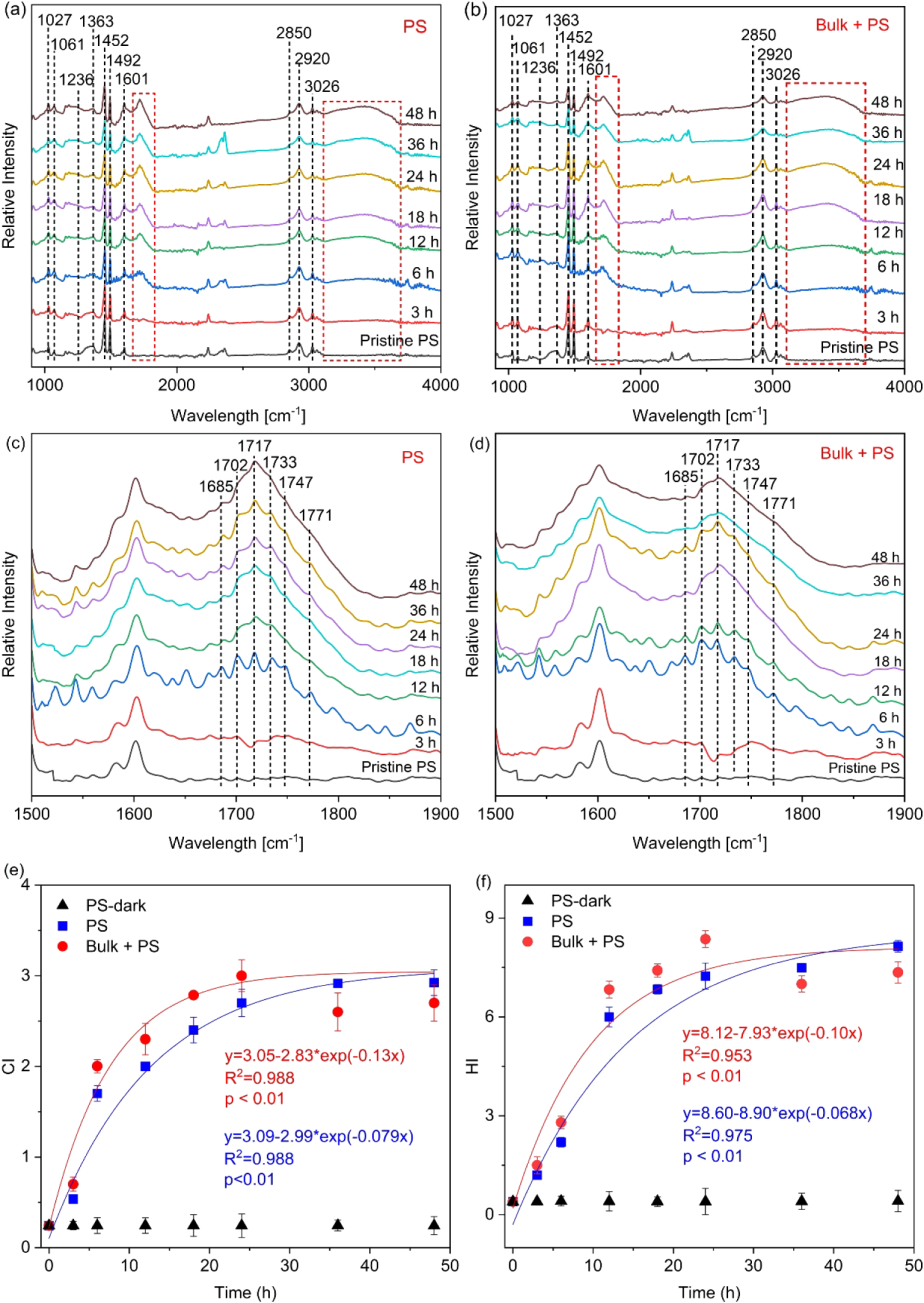
FTIR spectra of pristine and photoaged PS MPs at different exposure times, in the absence (a) and presence (b) of bulk DBC. Panels (c) and (d) offer detailed zoom-ins on the carbonyl region. Variations of carbonyl index (CI) (e) and hydroxyl index (HI) (f) value for PS MPs at different exposure times in the absence and presence of bulk DBC. Reaction conditions: 2.5 g/L PS MPs, 5 mg C/L bulk DBC, 2.5 mM phosphate buffer (pH = 7 ± 0.2), 1000 W mercury lamp light source.

Functional groups’ variations on PS MPs over time were tracked using FTIR spectroscopy. Pristine PS MPs displayed characteristic peaks across several wavenumbers: 1027, 1061, 1236, 1363, 1452, 1492, 1601, 2850, 2920, and 3026 cm^–1^ (Figure 1a). Specifically, the peaks at 1027 and 1061 cm^–1^ were attributed to C–H in plane bending/aromatic C–H bending.^41,42^ The 1363 cm^–1^ peak signalized C–H skeleton vibration and deformation,^43,44^ while the 1452 cm^–1^ peak was indicative of aromatic ring movement or −CH_2_– vibration.^41,42^ The bands at 2920 and 2850 cm^–1^ represented C–H stretching vibration of the CH_2_ and CH groups in the main PS chain.^42,45−47^ Peaks at 1492 and 1601 cm^–1^ were associated with C=C vibrations in aromatic rings.^41,42,48,49^ The 3026 cm^–1^ peak signified C–H vibrations within aromatic rings.^48,50^ As the photodegradation process progressed, a noticeable decrease in the intensity of peaks at 1363, 1452, 1492, 2850, and 3026 cm^–1^ was observed, whereas the 2920 cm^–1^ peak exhibited relative stability, indicating that photodegradation impacted both the fatty chains and the benzene rings, while the methylene groups within the main chain remained comparatively unaffected. Notably, the intensity at 1601 cm^–1^ increased with the aging time, which was likely due do the formation of new C=C unsaturated bonds within the fatty chains.^51^ Postphotoaging alterations were also evident in the peak shapes around 1027, 1061, and 1236 cm^–1^, pointing to the breakdown of the original C–H bonds/aromatic rings and the emergence of C–O bonds.^52^ Newly appearing absorption peaks at approximately 1717, and 3393 cm^–1^, after photoaging, were attributed to the stretching vibrations carbonyl (C=O), and hydroxyl (−OH) groups, respectively.^25,48,53^ A detailed analysis of the broad carbonyl region (Figure 1c) uncovered a variety of degradation products in aged PS MPs, including acetophenone, benzaldehyde, and acetic/formic acids (−CH_2_COOH), along with σ-lactone/benzoic acid, – CH_2_COOH, and benzoic anhydride, which manifested at 1685, 1702, 1717, 1733, 1747, and 1771 cm^–1^, respectively.^54−56^

### 3.2 DBC Accelerates the Photodegradation of PS MPs

As depicted in Figure S5, the presence of bulk DBC was observed to slightly intensify the yellowing effect of PS MPs compared to those aged in its absence. Furthermore, SEM images demonstrated that the presence of bulk DBC also made the surface of MPs smooth, but no obvious difference was observed compared to PS MPs alone (Figure S6). Additionally, size analysis indicated a more pronounced reduction in the average size of PS MPs photoaged with DBC compared to those without DBC (214 μm vs233 μm, *p* < 0.05, Table S1). These findings collectively indicated that DBC might accelerate the photodegradation of PS MPs.

To quantitatively assess the degradation extent of PS MPs, the carbonyl index (CI) and hydroxyl index (HI) (which are the ratios of the absorbance of the carbonyl peak at 1660–1850 cm^–1^ or hydroxyl peak at 3120–3710 cm^–1^ to the reference peak at 2870–2980 cm^–1^) were calculated as detailed in Text S3. For the control group (PS-dark), there was no significant increase in CI (*p* > 0.05), suggesting minimal degradation of the MPs in the absence of UV irradiation, as illustrated in Figure 1e. For the photoaging groups, the relationship between CI value and aging time was well-described by exponential equations (*r*^2^ > 0.97, *p* < 0.01), revealing an initial sharp rise of the CI value that gradually levels off, indicating a deceleration in the rate of CI increase. Notably, a plateau in CI values was reached in the PS MPs system, suggesting that the increase in carbonyl content continued until the surface of PS MPs became saturated.^57^ With the addition of bulk DBC, the CI values exhibited a quicker rise in the initial phase, peaking at 24 h with a value of 3.0 ± 0.18. This represented an 11% increase compared to PS MPs aged solely (CI = 2.7 ± 0.15), indicating that DBC accelerated the production of the carbonyl functional groups. However, a decline to a CI value of 2.6 ± 0.21 was observed at 36 h, before rising again to 2.7 ± 0.20 at 48 h. This pattern can be explained by the initial increase in carbonyl content until the surface reached saturation. The drop at 36 h suggested the exposure of a fresh surface layer that subsequently aged, leading to later increase in CI. Meanwhile, appeared to accelerate the production of the hydroxyl groups, as indicated by higher HI values in the presence of bulk DBC (Figure 1f), though this result was not statistically significant (*p* > 0.05). The accelerated photodegradation of PS MPs by bulk DBC was also observed under 30-days natural sunlight exposure (HI = 1.63 ± 0.24 for Bulk + PS vs 1.12 ± 0.18 for PS, Text S5, Figure S9). The acceleration of MPs’ photodegradation has also been documented in the presence of DOM, encompassing a range of DOM types such as FA/HA, sediment-extracted DOM, MPs-derived DOM, and oxalate.^31,36,37,58,59^ The enhancement was largely ascribed to the photosensitization properties of DOM. Notably, DBC has been identified as possessing superior photosensitization capabilities compared to many well-studied DOM, attributed to its smaller molecular size and numerous carbonyl compounds.^8^ As a photosensitizer, DBC has been reported to expedite the phototransformation of organic pollutants, including imidacloprid, 17β-estradiol, and chlortetracycline.^8,11,15^ In this study, the accelerated photodegradation of PS MPs in the presence of DBC was likely due to its photosensitivity, which might be related to its molecular size and chemical properties.

### 3.3 Effects of DBC Fractions

Following a stepwise fractionation process, bulk DBC was divided into three fractions based on molecular size: < 3 kDa, 3–30 kDa, and 30 kDa-0.45 μm, with a loss of 4% of the DOC. The fraction <3 kDa was identified as the most prevalent, accounting for 48% of the total DOC (Table S2). The intermediate group, 3–30 kDa, represented 30%, while the largest molecules, 30 kDa-0.45 μm, constituted 18%. Similar to NOM, the UV–vis spectral analysis of the DBC fractions revealed broad spectra with a decline across the spectrum and without clear peaks (Figure S10).^60^ The presence of a high absorption rate at shorter wavelengths (200–360 nm) suggested the fractions were rich in aromatic compounds.^61^ Additionally, the SUVA_254_ value (UV_254_ absorption relative to DOC), commonly used to assess aromatic content, showed an increase as MW increased (Table S2). This pattern implied that DBC with high molecular weight (HMW) exhibited more pronounced aromaticity, in agreement with the observations made by Tian et al.^11^ The *E*_2_/*E*_3_ value, defined as the ratio of UV absorbances at 250 and 365 nm, inversely correlated with molecular size or aromaticity (Table S2).^13,62^ The fluorescence intensities of DBC fractions were illustrated by the segmented EEM maps in Figure S11. Each fraction of DBC displayed relatively pronounced response intensities within regions III and V, indicating that humic and fulvic substances predominantly constituted their components.^63^ Given that the fluorescence intensity of the organic matter is primarily attributed to carboxyl groups,^64^ the higher fluorescence in lower MW DBC implied more abundance of carboxylic contents.^65,66^

**Figure 2:**
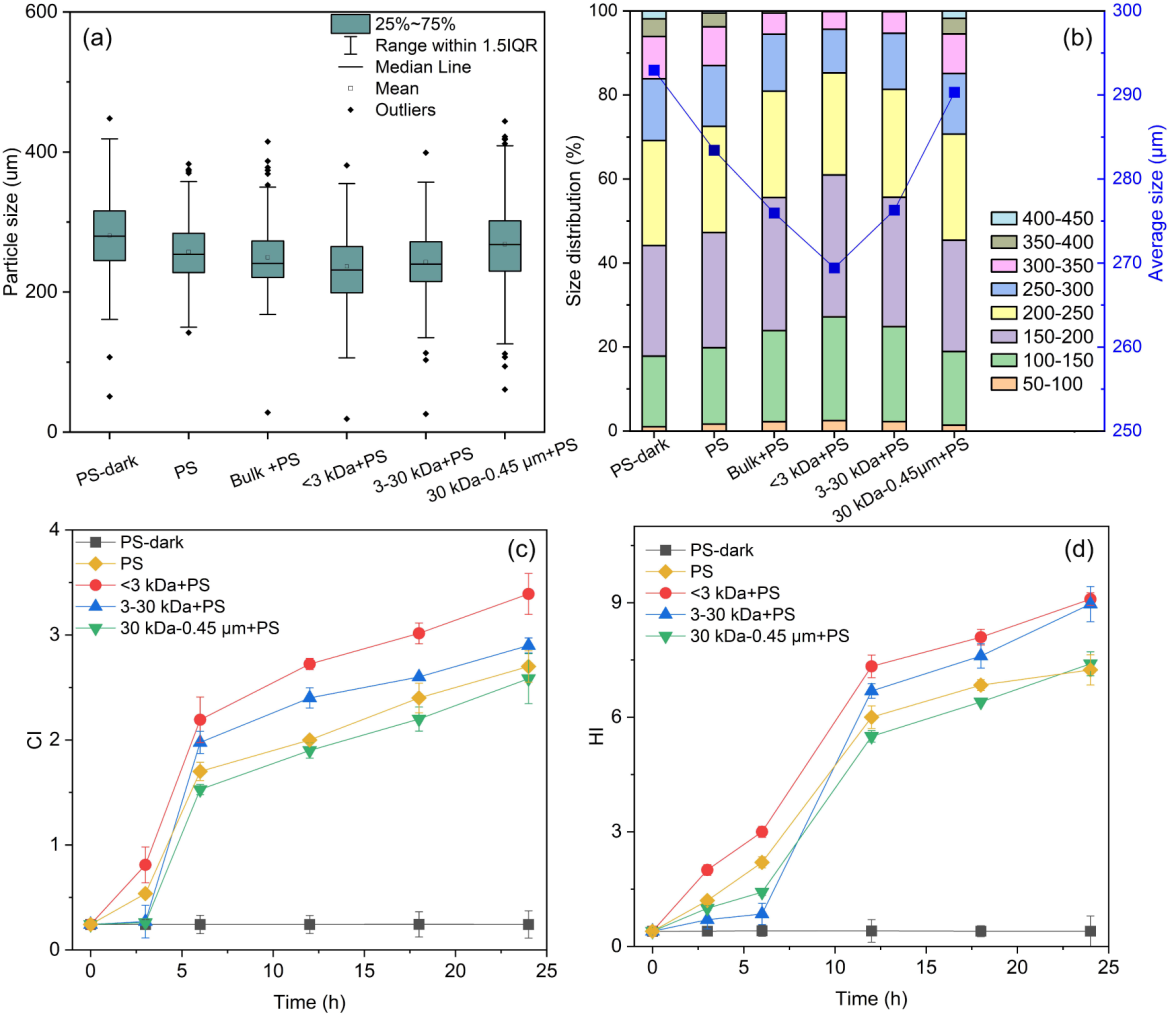
Size distribution and average sizes of unaged and aged PS MPs after irradiated for 24 h. (a) Data obtained from SEM images and calculated using ImageJ software. (b) Data obtained from LPSA (laser particle size analyzer). Variations of CI (c) and HI value (d) for PS MPs in the absence and presence of DBC fractions at different exposure times.

The influence of DBC fractions on the photodegradation of PS MPs was further investigated. PS MPs with low molecular weight (LMW) DBC (<3 kDa) seemed to undergo a more rapid shift in color from white to yellow (Figure S5). Additionally, PS MPs in the presence of the DBC fraction <3 kDa experience faster fragmentation compared to those mediated by other fractions (Figure 2a). Specifically, the average sizes of PS MPs were recorded as 237 μm, 243 μm, and 268 μm for the <3 kDa + PS, 3–30 kDa + PS and 30 kDa-0.45 μm + PS systems, respectively, following 24 h of irradiation. The LPSA analysis of PS MPs indicated that the average diameters changed from a pristine 293 μm to 283, 269, 276, and 290 μm for PS, < 3 kDa + PS, 3–30 kDa + PS, and 30 kDa-0.45 μm + PS systems, respectively, after 24 h of aging (Figure 2b). These values were larger than those obtained from SEM tests. This discrepancy was likely related to the aggregation of MPs and the water layer adsorbed on the MPs’ surface.^40,67^ The size and frequency distribution data of PS MPs confirmed that the photoaging induced size reduction, especially with the mediation of the <3 kDa fraction (Figure S12). These suggested that LMW DBC might accelerate the photodegradation process of PS MPs, but this effect was not statistically significant (*p* > 0.05).

Figure 2 illustrates the impact of different DBC fractions on CI and HI values. In the presence of the <3 kDa fractions, CI values were recorded at 3.39 ± 0.20, surpassing the value for individual PS MPs (2.70 ± 0.15). A similar trend was noted for the HI values (9.10 ± 0.16 for <3 kDa + PS, and 7.24 ± 0.40 for PS), suggesting that LMW DBC (i.e., < 3 kDa) fractions expedited the photoaging process of PS MPs, with statistically significant differences for both CI and HI (*p* < 0.05). Conversely, HMW DBC (i.e., 30 kDa-0.45 μm) inhibited the photodegradation of PS MPs, evidenced by significantly lower CI values (*p* < 0.05) and marginally lower HI values (*p* > 0.05) compared to PS MPs alone. Thus, the MW distribution of DBC influenced the photoaging degree of coexisting PS MPs. Tian et al. also observed an inverse correlation between the efficiency of DBC in enhancing chlortetracycline photodegradation and its MW; LMW DBC (<1 kDa) exhibited the stronger photosensitization ability due to abundant carbonyl compounds.^11^ Similarly, Awfa et al. reported that DOM with greater aromaticity but fewer carbonyl and/or carboxylic groups inhibited the photodegradation of carbamazepine more effectively.^68^ In this study, the distinct molecular properties of different MW fractions of DBC, such as aromaticity and carbonyl groups, determined their distinct role in the photodegradation of PS MPs.

### 3.4 2D-COS Analysis

2D-COS analysis was employed to investigate the sequential alterations in chemical bonds during the photodegradation of PS MPs. The synchronization diagram (Figure S15) revealed positive cross-peaks across nearly all functional groups, indicating concurrent changes throughout the aging process. Specifically, seven primary peaks appearing along the diagonal were located at: 1363, 1452, 1492, 1601, 1717, 2850, 3026, and 3393 cm^–1^. The sequential changes in functional groups of PS MPs in the absence of DBC (Tables 1 and S3–S7), progressing as the order: 3026 cm^–1^ (aromatic C–H) > 1492 cm^–1^ (aromatic ring) > 1452 cm^–1^ (aromatic ring or −CH_2_) > 2850 cm^–1^ (aliphatic −CH−) > 3393 cm^–1^ (−OH) > 1363 cm^–1^ (aliphatic −CH−) > 1601 cm^–1^ (aliphatic C=C) > 1717 cm^–1^ (C=O). The earlier reduction observed at 3026, 1492, and 1452 cm^–1^, compared to 2850 and 1363 cm^–1^, implied that the photodegradation of PS MPs primarily took place in the aromatic rings rather than the aliphatic segments. This observation aligned with previous research indicating that cleavage of the phenyl ring C–C bonds predominated in the degradation of PS MPs under high-energy UV exposure.^69^ However, it contradicted the popular hypotheses in many studies that UV-induced degradation began with C–H bond scission.^53,70,71^

**Table 1:** Functional Group Change Sequence of the PS MPs in the Absence and Presence of DBC Based on 2D-COS Analysis^a^

	sequence of functional groups
PS	3026 > 1492 > 1452 > 2850 > 3393 > 1363 > 1601 > 1717
bulk + PS	1492 > 1452 > 3026 > 2850 > 1363 > 1717 > 1601 > 3393
<3 kDa + PS	3026 > 1492 > 1452 > 2850 > 1363 > 1717 > 1601 > 3393
3–30 kDa + PS	1492 > 1452 > 3026 > 2850 > 1363 > 1717 > 1601 > 3393
30 kDa-0.45 μm + PS	1492 > 1452 > 3026 > 2850 > 1363 > 1717 > 1601 > 3393

a Note. 3026 cm^–1^ (aromatic C–H), 1492 cm^–1^ (aromatic ring), 1452 cm^–1^ (aromatic ring/-CH2), 2850 cm^–1^ (aliphatic −CH−), 3393 cm^–1^ (−OH), 1363 cm^–1^ (aliphatic −CH−), 1601 cm^–1^ (aliphatic C=C), 1717 cm^–1^ (C=O).

The 2D-COS revealed that the degradation sequence of 3026, 1492, and 1452 cm^–1^ remained occurring at the forefront in the presence of DBC fractions (Tables 1 and S3–S7), indicating that DBC did not influence the preference for aromatic rings. However, the 2D-COS analysis revealed a different sequence of peaks for C=O (1717 cm^–1^), C=C (1601 cm^–1^), and −OH (3393 cm^–1^) compared to the sole PS MPs system. Specifically, upon the addition of DBC fraction to the system, it was observed that the C=O peak formed rapidly initially, followed by the emergence of the C=C and −OH peaks. A similar phenomenon was observed by Yu et al., where the C=O functional group of PS MPs changed before −OH in the presence of Cu^2+^ and Pb^2+^ due to the generation of a large number of •OH.^72^

### 3.5 Mechanism of DBC-Mediated Photodegradation of MPs

#### 3.5.1 Screening Effects of Different DBC Fractions

Due to the presence of chromophores such as carbonyl groups and aromatic compounds, DBC can act as optical filter, potentially interfering with the photolysis of PS MPs.^30^ The concept of the light screening factor (*S*_λ_) (Text S4) was introduced to gauge the impact of DBC as a filtering agent. *S*_λ_ equals 1 when no light attenuation occurs. As illustrated in Table S2, the *S*_λ_ of DBC fraction-containing solution increased with the decrease in DBC fraction’s MW, indicating that DBC with higher MW had a stronger light screening effect, which aligned with findings from other studies.^15,73^ The phenomenon could be attributed to the abundant aromatic components (higher SUVA_254_ and lower *E*_2_/*E*_3_) present in HMW DBC. Thus, the presence of DBC, especially HMW DBC fractions, might inhibit the direct photolysis of PS MPs by competing for light and photons.^15,73^

#### 3.5.2 Roles of Reactive Intermediates

**Figure 3:**
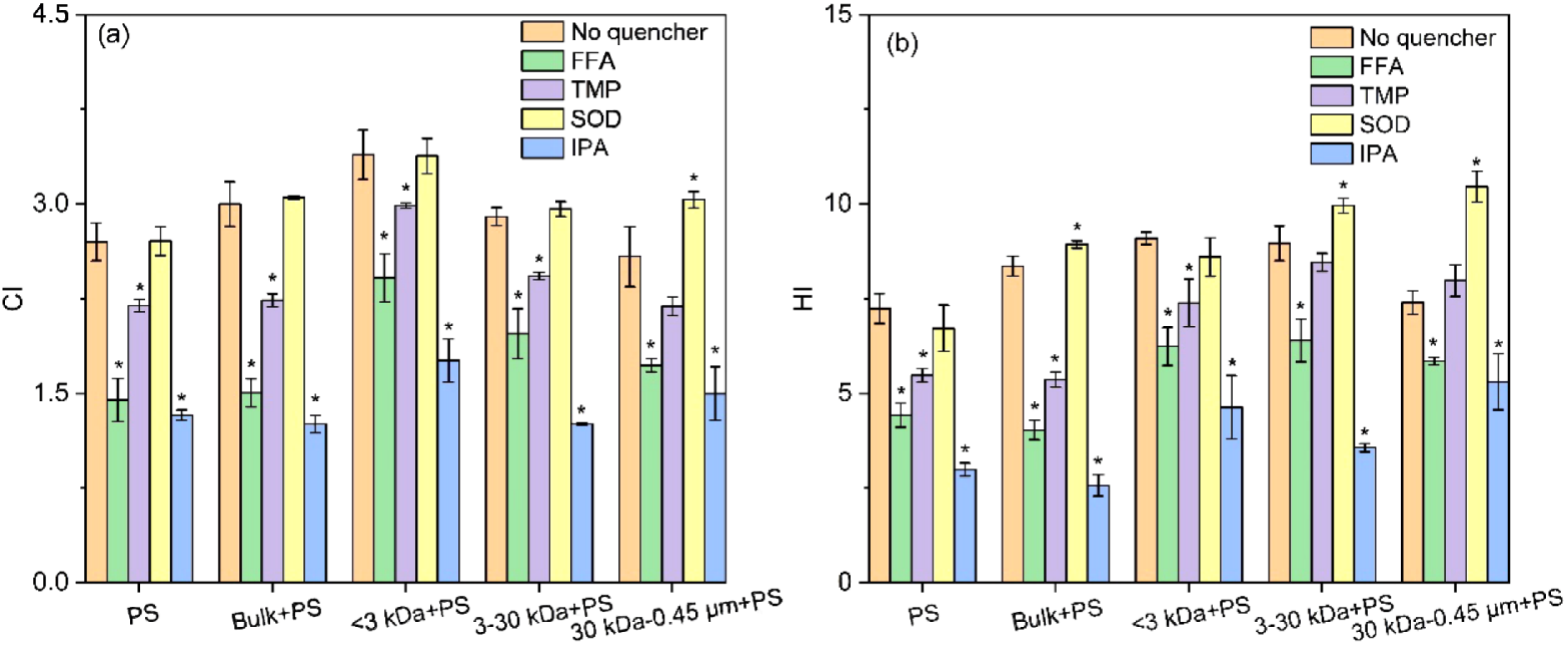
CI (a) and HI values (b) of PS MPs at the 24 h time point in the absence or presence of bulk DBC/DBC fractions, with and without the addition of reactive intermediate (RI) scavengers (FFA, TMP, SOD, IPA). The scavengers were replenished every 12 h to counteract their depletion. Asterisks indicate statistically significant differences (*p* < 0.05) compared with their corresponding no quencher group based on *t* test.

The clarification of RIs’ role in transforming PS MPs was achieved by conducting quenching experiments with various scavengers and evaluating the changes in CI and HI values with or without their addition. As demonstrated in Figure 3, the addition of IPA, FFA and TMP, which scavenge •OH, ^1^O_2_, and ^3^PS*, respectively, lowered CI/HI values within the PS MPs system. Specifically, CI/HI values decreased by 51%/59%, 46%/39% and 19%/24% with the addition of IPA, FFA and TMP, respectively. These reductions highlight the critical role of •OH, ^1^O_2_ and, ^3^PS*, particularly •OH (*p* < 0.05) and ^1^O_2_ (*p* < 0.05), in the photodegradation of PS MPs. •OH and ^1^O_2_ have been reported to mediate the photodegradation of PS MPs in previous studies due to their strong oxidation potentials.^39,53^ The addition of SOD, a quencher of O_2_•^–^, had no effect on the CI/HI values in PS MPs system (*p* > 0.05), implying that O_2_•^–^ played a negligible role in the photodegradation of PS MPs in this context. In the presence of bulk DBC and DBC fractions, the superior role of •OH and ^1^O_2_ in the photodegradation process of PS MPs over ^3^DBC*/^3^PS* was also observed. Nevertheless, after scavenging O_2_•^–^, CI and HI values increased in the systems with 3–30 kDa and 30 kDa-0.45 μm. Particularly, the scavenging of O_2_•^–^ led to an increase of 17% in CI and 41% in HI in the system within 30 kDa-0.45 μm DBC + PS system. It suggested that O_2_•^–^ likely inhibited the photodegradation of PS MPs in the presence of relatively high MW DBC fractions. O_2_•^–^, with an unpaired electron, typically acts as a more efficient reducing agent than an oxidizing one, evidenced by its standard potential (E_0_(O_2_/O_2_•^–^) = −0.33 V).^74,75^ In this study, the suppression of PS MPs photodegradation by O_2_•^–^ might be attributed to its function as an electron donor, rapidly converting the initial photodegradation product of PS MPs back into its original state.^8,76^ Similar findings have been reported in previous studies. For instance, the phototransformation of acetaminophen exhibited notably faster rates in solutions containing DOM when SOD was introduced.^76^ Similarly, the addition of SOD resulted in an enhanced phototransformation rate of 17β-estradiol in the presence of bamboo-derived DBC.^8^

**Figure 4:**
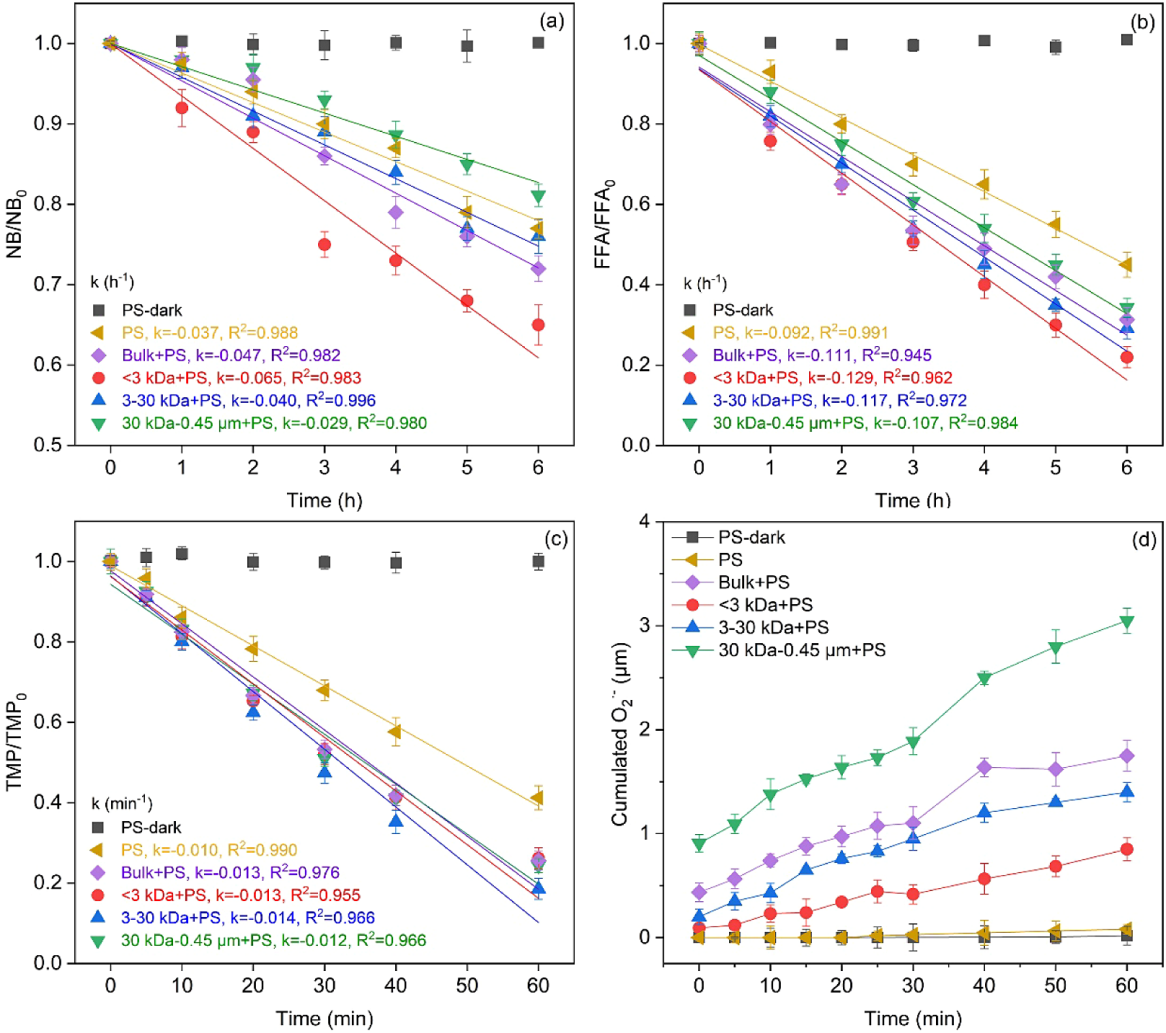
Formation capacity of RIs for PS MPs under various conditions: (a) •OH, (b) ^1^O_2_, (c) ^3^DBC*/^3^PS*, and (d) O_2_•^–^ in dark conditions or in the absence and presence of bulk DBC- and DBC fraction-containing aqueous solutions during irradiation.

To validate the quenching experiments, the production of •OH, ^1^O_2_, ^3^DBC*/^3^PS* and O_2_•^–^ by PS MPs and DBC was quantified using chemical probe techniques. The depletion rate of NB in the sole PS MPs system, represented by a first-order rate constant (k_NB–•OH_), was measured at 0.037 h^–1^, slower than in the <3 kDa + PS (0.065 h^–1^) and 3–30 kDa + PS (0.040 h^–1^) systems but faster than in 30 kDa-0.45 μm + PS system (0.029 h^–1^) (Figure 4a). The steady-state concentrations of •OH ([•OH]_ss_) were evaluated using the second-order rate constant of NB and •OH (3.9 × 10^9^ M^–1^ s^–1^ (k_NB–•OH_)) and the pseudo-first-order rate constant (k_NB′_).^77,78^ The PS MPs system showed a [•OH]_ss_ of 2.62 × 10^–15^ M, whereas the systems <3 kDa + PS, 3–30 kDa + PS and 30 kDa-0.45 μm + PS exhibited [•OH]_ss_ of 4.64 × 10^–15^, 2.85× 10^–15^ and 2.05 × 10^–15^ M, respectively. This suggested LMW DBC (i.e., < 3 kDa) markedly boosted [•OH]_ss_, whereas HMW DBC (i.e., 30 kDa-0.45 μm) led to a decrease in [•OH]_ss_. Some studies also indicated that LMW DOM was most efficient in producing •OH,^79−82^ although the specific relationship with molecular properties remains debated (discussed in 3.5.3). While all DBC fractions accelerated the production of the steady-state concentrations of ^1^O_2_ ([^1^O_2_]_ss_) (Figure 4b), this effect increased as MW decreased. The production of ^1^O_2_ by the photosensitivity of DOM was reported to be positively associated with the *E*_2_/*E*_3_ value and carbonyl-containing structures of DOM.^12,79,83^ In this study, the higher *E*_2_/*E*_3_ value and the presence of more abundant carbonyl functional groups in lower MW DBC might contribute to higher [^1^O_2_]_ss_, thereby accelerating the photodegradation of MPs to a greater extent. After 60 min of UV exposure, TMP degradation rates in the presence of various DBC fractions were higher compared to PS MPs alone (Figure 4c), indicating additional ^3^DBC*/^3^PS* generated in the presence of DBC fractions. Herein, the concentrations of triplet species, including ^3^PS*, ^3^PS-DOM*, and ^3^DBC*, were not calculated due to the lack of data on the second-order rate constant between ^3^PS*/^3^PS-DOM* and TMP. Despite this, faster TMP degradation in the presence of LMW DBC suggested that more ^3^DBC*/^3^PS* was generated. In addition, O_2_•^–^ was hardly produced from the sole PS MPs as evidenced by scant XTT formazan formation (Figure 4d). Notably, slight cumulative concentration of O_2_•^–^ were observed in the <3 kDa + PS (0.85 ± 0.11 μM) and 3–30 kDa + PS (1.40 ± 0.09 μM) systems, while 30 kDa-0.45 μm + PS displayed a substantial cumulative concentration of O_2_•^–^ (3.05 ± 0.12 μM). The phenolic groups were previously identified as active moieties in DOM for O_2_•^–^ generation.^79^ HMW DBC with more amount of aromatic structure (as evidenced by SUVA_254_ and *E*_2_/*E*_3_) might contain more aromatic phenolic moieties, which could be responsible for more O_2_•^–^ generation than LMW DBC.^12^

In summary, •OH, ^1^O_2_, and, ^3^DBC*/^3^PS* were identified as key RIs in accelerating the photodegradation of PS MPs with their contributions ranked in the order •OH > ^1^O_2_ > ^3^DBC*/^3^PS*, while O_2_•^–^ was found to be unfavorable for this process. The presence of bulk DBC promoted the production of •OH, ^1^O_2_, and ^3^DBC*/^3^PS*, facilitating the photodegradation of PS MPs. The photosensitivity production of •OH, ^1^O_2_ and O_2_•^–^ were associated with MWs of DBC. Importantly, LMW DBC (i.e., < 3 kDa and 3–30 kDa) contained more carboxyl groups which accelerated the degradation of PS MPs to a larger extent due to their ability to generate more •OH and ^1^O_2_. Nevertheless, HMW DBC (30 kDa-0.45 μm), characterized by its high aromaticity and low carbonyl groups, not only scavenged •OH but also produced abundant O_2_•^–^, thereby inhibiting the photodegradation of PS MPs. Overall, the prevalence of LMW DBC within bulk DBC was paramount in its capacity to enhance the photodegradation of PS MPs through its photosensitivity process.

#### 3.5.3 Pathways of Photodegradation of PS MPs

**Figure 5:**
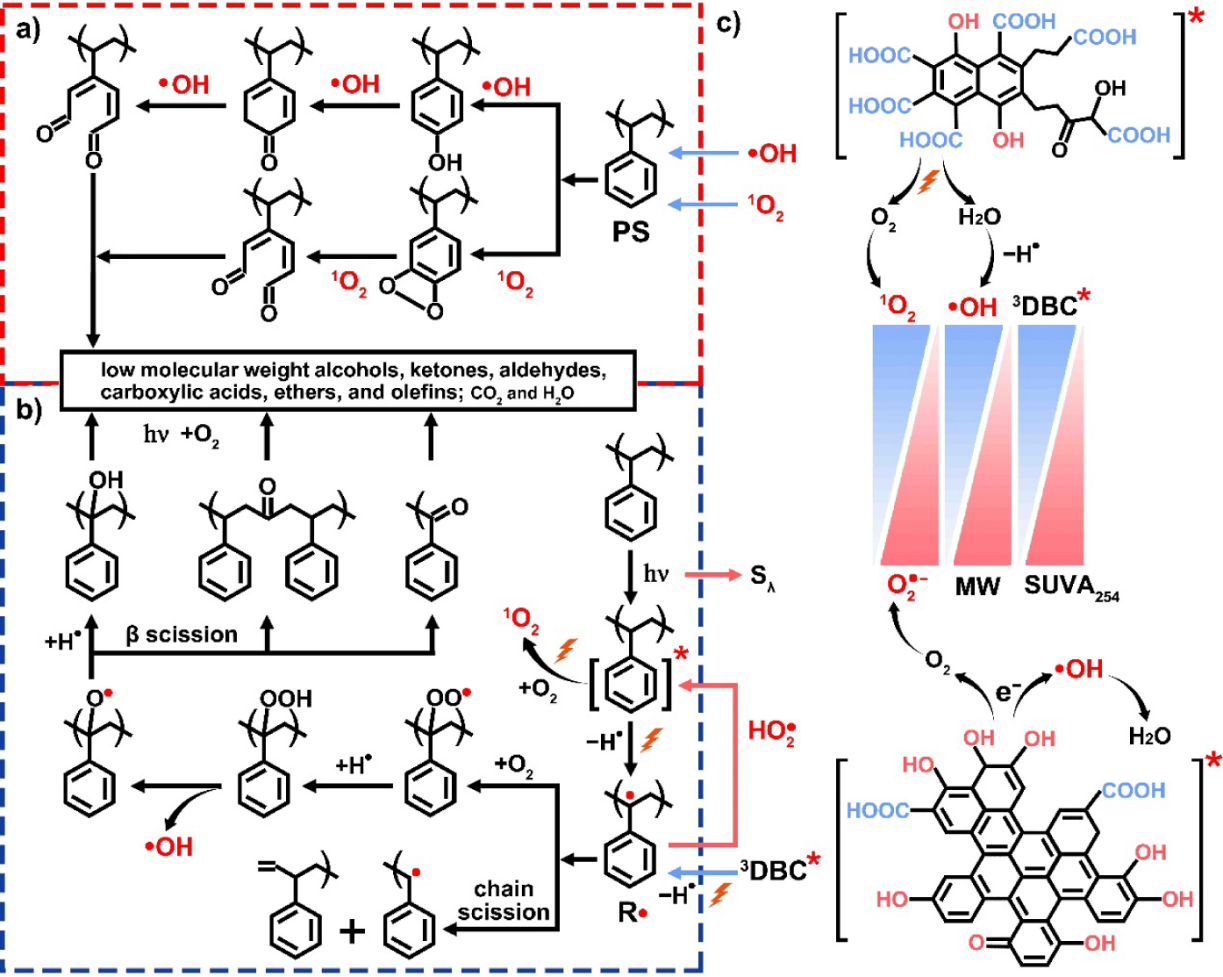
Proposed photodegradation mechanisms of PS MPs. (a) Preferential attack of the benzene ring by •OH and ^1^O_2_ both in the absence and presend of bulk DBC/DBC fractions. (b) Traditional photodegradation pathway of aliphatic segments. (c) Mechanism of DBC fraction-mediated photodegradation.

As elucidated in 2D-COS analysis, the photodegradation of PS MPs is likely to occur primarily in the aromatic rings rather than in the aliphatic segments. The primary degradation of aromatic rings is likely facilitated by the fast attack of ^1^O_2_ and •OH (Figure 5a). The electrophilic ^1^O_2_ and •OH preferentially react with the benzene ring due to its high electron density.^53,84^ As demonstrated in previous studies, ring addition of •OH strongly prevails over hydrogen abstraction in the cases of aromatic compounds.^85−87^ The •OH addition could produce phenyl −OH (3436–3450 cm^–1^) which might further transform into aldehyde in the presence of excessive •OH.^53,88,89^ The addition of ^1^O_2_ on benzene ring might generate hydroperoxide groups (-OOH, 3220 cm^–1^) or endoperoxide intermediates (O–O),^84,90,91^ and then further form aldehyde in the presence of excess ^1^O_2_.^53,90^

The degradation of aliphatic segments, such as −CH (1363 and 2850 cm^–1^), might follow a traditional degradation pathway (Figure 5b). The transfer of the energy from ^3^PS* to the C–H bond can generate alkyl radicals (R•). R• can react with O_2_ to form peroxyl radicals (ROO•), which in turn abstract hydrogen from other chains and form R–OOH (3220 cm^–1^). Subsequently, the R–OOH groups could further transform into relatively stable products such as alcohols (3393 cm^–1^), ketones (1685 cm^–1^), aldehydes (1702 cm^–1^), carboxylic acids (1717 and 1747 cm^–1^), and olefins (1601 cm^–1^) through processes such as hydrogen abstraction, β-scission, and Norrish I and II reactions.^71,92^ Alternatively, R• may undergo disproportionation, resulting in chain scission and the formation of olefins (1601 cm^–1^), as evidenced by the increase in C=C groups.^92,93^ According to the 2D-COS analysis, the occurrence of 1601 cm^–1^ (C=C) before 1717 cm^–1^ (C=O) suggested that the R• primarily underwent chain scission rather than oxidative degradation by oxygen. Meanwhile, excessive ^1^O_2_ and •OH might also mediate the phototransformation of the C–H bond of PS MPs. For example, •OH or ^1^O_2_ might abstract hydrogen to generate R•.^53,71^ The unsaturated olefins likely underwent similar transformation within the benzene ring with the addition of •OH or ^1^O_2_, and produce −OH or O–O and C=O.^85,94^

The photodegradation of PS MPs mediated by DBC was elucidated through the generation and mediating mechanisms of RIs derived from DBC. As discussed in 3.5.2, LMW DBC (i.e., < 3 kDa and 3 kDa-30 kDa) promoted the generation of ^1^O_2_, •OH and, ^3^DBC*/^3^PS*, while HMW DBC (i.e., 30 kDa-0.45 μm) scavenged •OH and generated more O_2_•^–^. Typically, ^1^O_2_ could be generated by the energy transfer of ^3^DBC* to O_2_, which is positively correlated with *E*_2_/*E*_3_ and negatively correlated with SUVA_254_.^81,82^ The generation of O_2_•^–^ was due to the electron transfer of ^3^DBC* to O_2_, thus HMW DBC with abundant reductive aromatic phenols produced more O_2_•^–^.^95^ Notably, various mechanisms contribute to the photogeneration of •OH by DOM.^95^ One well-documented mechanism involves the reduction of O_2_ by ^3^DOM* to generate O_2_•^–^, which then undergoes dismutation to yield hydrogen peroxide (H_2_O_2_), ultimately leading to the formation of •OH.^96^ Besides, ^3^DOM* may directly generate •OH by abstracting hydrogen from H_2_O.^80,82^ Herein, [•OH]_ss_ generated in the presence of DBC fractions followed similar trends with [^1^O_2_]_ss_ rather than [O_2_•^–^]_ss_.^96^ Moreover, the scavenging of O_2_•^–^ increased the photodegradation of PS MPs, indicating that •OH was more likely generated from hydrogen abstraction by ^3^DBC* rather than O_2_•^–^.^95^ However, the scavenging phenomenon of •OH by HMW DBC was likely due to the electron transfer or scavenging from the reductive aromatic phenols within HMW DBC (Figure 5c).

As discussed in 2D-COS, C=O formed before C=C and −OH, the rapid formation of C=O was related to the direct addition of abundant ^1^O_2_ and •OH on the benzene ring and unsaturated olefins, accelerating the conversion of phenolic −OH or olefins and the production of aldehydes. Apart from serving as a crucial precursor for •OH and ^1^O_2_, increased production of ^3^DBC*/^3^PS* may transfer more energy to the C–H bond of DBC/PS, generating more organic radicals and thereby enhancing the phototransformation of aliphatic components. In the case of HMW DBC, apart from scavenging •OH, the rise in O_2_•^–^ production was identified as a critical factor hindering the photodegradation of PS MPs. Herein, O_2_•^–^ probably functioned as an electron donor, inducing the reduction of R• to its original state, consequently impeding further degradation and reducing the formation of −OH, C=C, and C=O.^8,76^

## 4 Environmental Implications

Due to the growing prevalence of MPs in the environment and their long-term, widespread presence, the weathering issue of MPs has intensified. The photodegradation mechanism and mediated roles of environmental compunds in the photochemical weathering of MPs have been extensively studied.^25,26,30^ Traditional degradation pathways have suggested that photodegradation of PS MPs occurs primarily at the aliphatic segments, mediated by organic radicals and RIs.^71,97,98^ Our study indicated that the photodegradation of PS MPs initially occurred at the benzene ring due to the preferential attack of •OH and ^1^O_2_. Additionally, the negative role of O_2_•^–^ in the photodegradation of MPs was revealed, which contradicted findings from some previous studies.^37,70^ These findings offer new insights into the photodegradation mechanism of PS MPs.

DBC leached from pyrolyzed biomass exhibits higher resistance compared to other DOM, enabling it to actively participate in the biogeochemical processes of environmental pollutants.^8,99^ Due to the abundance in aromatic components and carbonyl groups, DBC exhibits strong photosensitizing capacity, mediating the phototransformation of many pollutants, such as organic pollutants,^8,11,100^ and heavy metals.^14,101^ Our study found that DBC leached from pyrolyzed wood residue facilitated the photodegradation of PS MPs, indicating its potential in mitigating the persistence of MPs in aquatic environments. It further highlights the molecular size as a key factor influencing the photosensitizing ability of DBC, revealing its relationship with the chemical properties such as aromatic phenols and carbonyl groups. These findings help to understand the photosensitizing process of DBC/DOM, as well as its role in mediating the degradation of pollutants. Natural events, such as wildfires or biomass burning, serve as significant sources of DBC input into aquatic systems.^102,103^ Consequently, the role of DBC may be particularly pronounced in waters affected by these events. Therefore, considering DBC-induced phototransformation is crucial when assessing the fate of MPs in environments affected by natural disturbances. Overall, these findings contribute to the understanding of the photochemical weathering of MPs and the potential environmental impacts of natural events.

## Supporting Information Available

Determination for the concentration of NB, FFA, TMP and XTT formazan (Text S1); calculation for the concentration of RIs (Text S2); Calculation for CI and HI (Text S3); calculation for *S*_λ_ (Text S4); Natural sunlight exposure of PS MPs (Text S5); Ultrafiltration process of DBC solution (Figure S1); The emission spectrum of the mercury lamp (Figure S2); Changes of EEM of bulk DBC and DBC fractions during irradiation time (Figure S3); Approaches for measuring PS MPs particle size using ImageJ software (Figure S4); Digital images of PS MPs with or without the mediation of DBC fractions at different exposure times (Figure S5); SEM images for PS MPs after 24 h photoaging with or without the presence of DBC (Figure S6); DOC concentration of solution during photoaging (Figure S7); Changes of EEM of PS MPs system during irradiation time (Figure S8); FTIR spectra of PS MPs under natural sunlight exposure with and without bulk DBC (Figure S9); UV–vis absorption spectra of 5 mgC/L DBC and DBC fractions in phosphate buffer (Figure S10); EEM maps of bulk DBC and DBC fractions (Figure S11); Hydrodynamic size and frequency distributions of PS MPs obtained by LPSA (Figure S12); The FTIR spectra of photoaged PS MPs, in the presence of DBC fractions (Figure S13); Formation capacity of (a) •OH, (b) ^1^O_2_, (c) ^3^DBC*/^3^PS*, and (d) O_2_•^–^ for bulk DBC and DBC fractions (Figure S14); Synchronous 2D-FTIR–COS maps of PS MPs under different incubation times with or without the addition of DBC (Figure S15); Asynchronous 2D-FTIR–COS maps of PS MPs under different incubation times with or without the addition of DBC (Figure S16); DOC change of bulk DBC or DBC fractions solution (Figure S17); Particle size of pristine and photoaged PS MPs after 48 h aging (Table S1); The characteristics of bulk DBC and DBC fractions (Table S2); 2D-COS Data on the assignment and sign of each cross-peak in synchronous and asynchronous maps of PS MPs (Table S3–S7) (PDF)
