# Supplementary material for: Dissolved Black Carbon Facilitates the Photodegradation of Microplastics via Molecular Weight-Dependent Generation of Reactive Intermediates
Source: Environ Sci Technol. 2024 Aug 12;58(34):15181–93. doi: 10.1021/acs.est.4c03831 (PMC11360373; doi:10.1021/acs.est.4c03831)
Supplement: Supplementary file 1 — es4c03831_si_001.pdf [file es4c03831_si_001.pdf]

Supplementary materials for:

Dissolved Black Carbon Facilitates the Photodegradation of Microplastics via Molecular Weight-Dependent Generation of Reactive Intermediates

Qin Ou [a, b, #] & Yanghui Xu [a, b, #], Xintu Wang [a, c], Jan Peter van der Hoek [b, e], Guo Yu [c], Gang Liu [a, d, *]

[a] Key Laboratory of Drinking Water Science and Technology, Research Centre for Eco-Environmental Sciences, Chinese Academy of Sciences, Beijing, 100085, P. R. China

[b] Section of Sanitary Engineering, Department of Water Management, Faculty of Civil Engineering and Geosciences, Delft University of Technology, Stevinweg 1, 2628 CN Delft, The Netherlands

[c] College of Environmental Science and Engineering, Guilin University of Technology, Guangxi, 541004, China

[d] University of Chinese Academy of Sciences, Beijing, China

[e] Waternet, Department Research & Innovation, P.O. Box 94370, 1090 GJ Amsterdam, the Netherlands

[#] Qin Ou and Yanghui Xu contributed equally to this manuscript

The supplementary material includes 5 texts, 17 figures, and 7 tables in 24 Pages.

Text S1. Determination for the concentration of NB, FFA, TMP and XTT formazan

During the irradiation, the remaining NB, FFA and TMP in the solutions was measured using HPLC with an Agilent 144 Eclipse XDB-C18 reversed phase column (5 µm × 250 mm × 4 mm). 60% acetonitrile: 40% ultrapure water (v:v) with a detection wavelength of 263 nm for NB;[1] 50% acetonitrile: 50% 0.1 wt% phosphoric acid (v:v) with a detection wavelength of 220 nm for FFA;[2] 60% acetonitrile: 40% 0.1 wt % phosphoric acid (v:v) with a detection wavelength of 220 nm for TMP.[3] The concentration of XTT formazan, the reaction product of XTT with $O_2^{\bullet-}$ under light irradiation, was determined using ultraviolet-visible spectrometer at 470 nm[4].

Text S2. Calculation for the concentration of RIs

The photo-transformation of furfuryl alcohol (FFA) can be expressed as[3]:

$$\frac{d[FFA]}{dt} = -\, k_{1O2,\ FFA}[FFA][1O2]ss$$

$$\frac{d[FFA]}{dt} = -\, k_{obs}[FFA]$$

Therefore, the steady steady-state concentration of $^1O_2$ ($[^1O_2]_{ss}$, M) can be determined as:

$$[1O2]ss = \frac{k_{obs}}{k_{1O2,\ FFA}}$$

Where k_{FFA}=1.2 × 10^8 M^{-1} S^{-1}.[5]

The steady-state concentrations of •OH ($[•OH]_{ss}$, M) were evaluated using the second-order rate constant of NB and •OH (3.9 × 10^9 M^{-1} s^{-1} ($k_{NB-•OH}$)) and the pseudo-first-order rate constant ($k_{NB'}$).[6, 7], expressed as:

$$[•OH]ss = \frac{k'_{NB}}{k_{NB-•OH}}$$

TMP reacts with both $^3DBC^*$ and $^3PS^*$ in solution. Therefore, the initial photo-transformation rate of TMP can be written as follows:

$$-\frac{d[TMP]}{dt} = k_{3DBC*,TMP}[TMP][3DBC*]ss + k_{3PS*,TMP}[TMP][3DBC*]ss$$

Due to the lack of data on the second-order rate constant between $^3PS^*$ and TMP ($k_{3PS*,TMP}$), further calculation of $[^3DBC^*]_{ss}$ and $[^3PS^*]_{ss}$ was not continue.

The cumulated concentration of $O_2•^-$ (M) was calculated as:[1, 8]

$$[O_2^{\bullet -}]ss = 2 \times \frac{A}{ab}$$

Where, a is the extinction coefficient of XTT formazan, 21,600 $M^{-1}cm^{-1}$;[4] b indicates the distance the light travels through the quartz cuvette, 1 cm for this experiment. A is the tested absorbance of the solution.

Text S3. Calculation for carbonyl index (CI) and hydroxyl index (HI)

Quantifying the results was crucial to delineate the photo-oxidation process of plastics. The CI and HI were introduced as key metrics. These indices represent the relative abundance of carbonyl and hydroxyl groups, respectively, increases in the CI and HI are associated with an increase in the polymers' surface oxidation state. The calculation of CI and HI were using the specific area under band (SAUB) methodology.[9, 10] The peaks were analysed without smoothening the data. Net peak heights were determined by subtracting the height of the baseline using integration method in Origin software.[11, 12] CI and HI are calculated by comparing the net heights of the created bands (1660-1850 cm^{-1} for CI and 3120-3710 cm^{-1} for HI) in the spectra to a reference peak. In this study, the 2870-2980 cm^{-1} band, corresponding to the C–H stretching vibration of the CH_2,[13, 14] was chosen as this reference, as it remains stable under UV irradiation. The CI and HI equations are as follows:

$$CI = A\ 1660\text{-}1850\ cm^{-1} / A\ 2870\text{-}2980\ cm^{-1}$$

$$HI = A\ 3120\text{-}3710\ cm^{-1} / A\ 2870\text{-}2980\ cm^{-1}$$

Where, A denotes the peak intensity.

Text S4. Calculation for light screening factor (S_λ)

In this context, the concept of the light screening factor (S_λ) is introduced to gauge the impact of DBC as a filtering agent, with its value derived through following calculation formula[1, 15]:

$$S_\lambda = \frac{1-(10^{-\alpha_\lambda l})}{2.303\alpha_\lambda l}$$

$$\alpha_\lambda = \frac{2.303 \times A_\lambda}{l}$$

$$S_{250-450} = (\int_{250}^{450} s_\lambda \, ds_\lambda)/(450-250)$$

where α_λ (cm^{-1}) represents the decadic specific absorption coefficient, and l (cm) indicates the distance the light travels through the quartz cuvette, set at 1 cm for this experiment, A_λ is the recorded absorbance at a designated wavelength. $S_{250-450}$ represents the average optical screening factor between 250 and 450 nm for DBC with composite light sources.

Text S5. Natural sunlight exposure of PS MPs

Natural sunlight exposure of PS MPs was conducted on the rooftop from August 4 to August 28, 2023, in Beijing. The CI was 0.50 ± 0.04 for PS MPs after natural weathering. The natural light weathering of PS MPs (2.5 g/L), with or without the addition of bulk DBC (5 mg C/L), was conducted, from May 19 to June 18, 2024, under the same conditions and in the same location. The results showed that the presence of bulk DBC slightly increased HI value of PS MPs (1.63 ± 0.24 vs. 1.12 ± 0.18). However, there was no obvious difference in the CI value between Bulk + PS (0.42 ± 0.06) and PS (0.40 ± 0.04). These experiments were conducted in duplicate, with the error bars representing the maximum and minimum values.

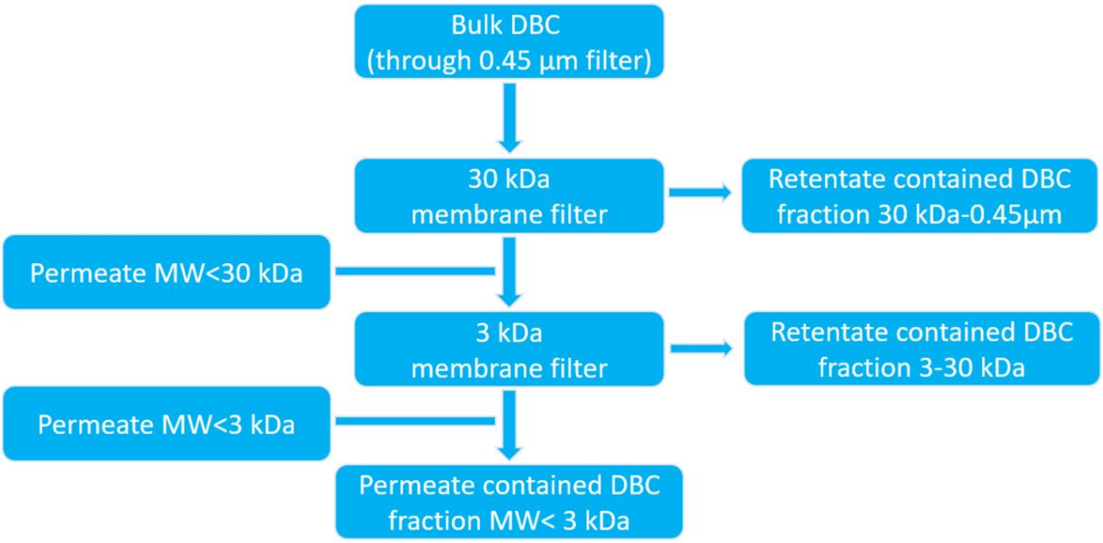

Figure S1. Ultrafiltration process of DBC solution[16].

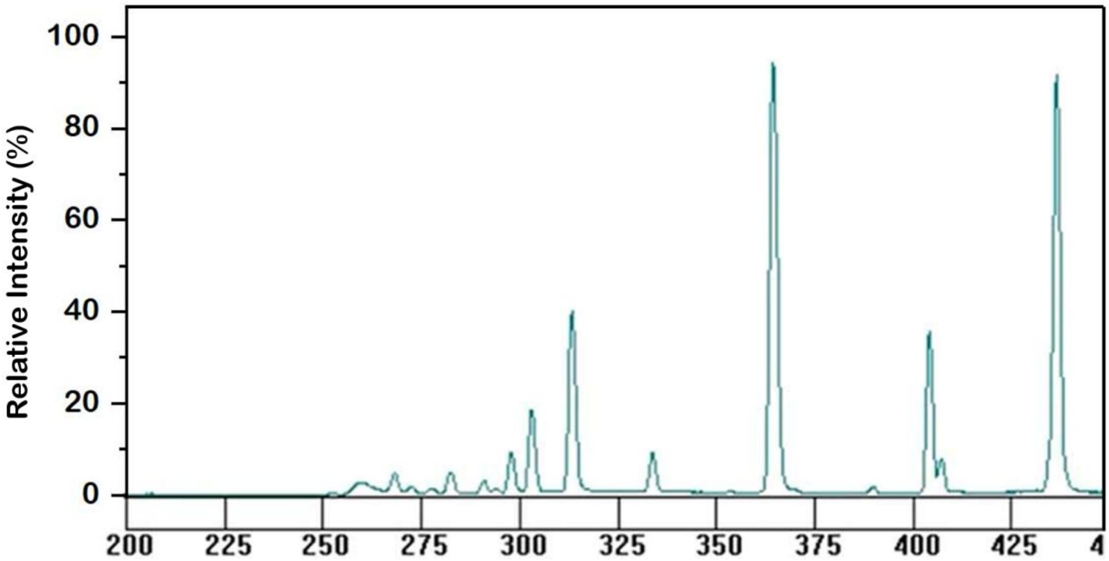

Figure S2. The emission spectrum of the mercury lamp (supplied by manufacturer)

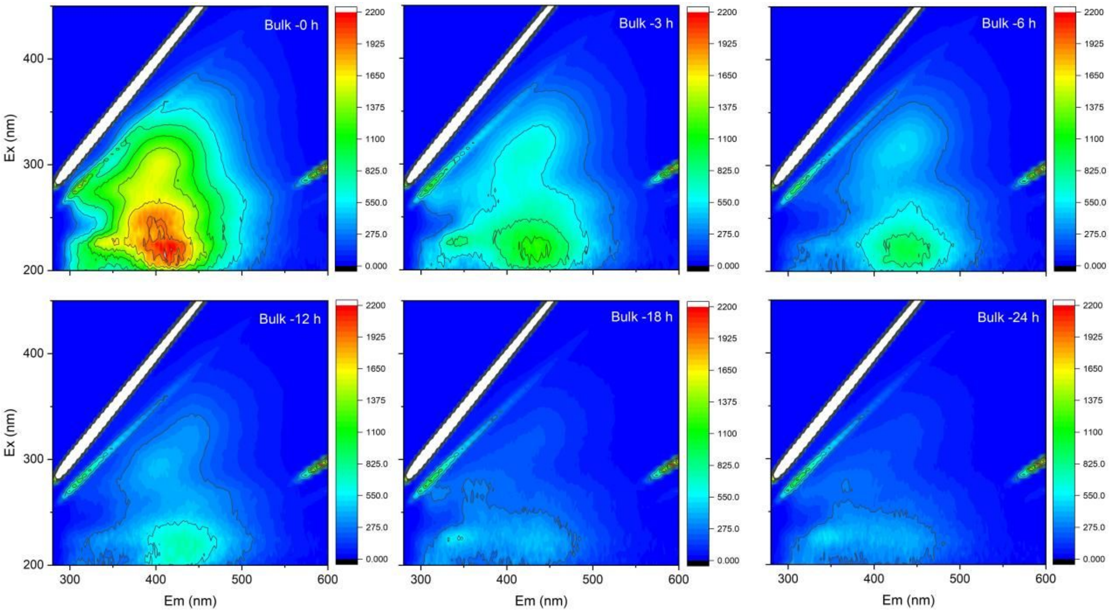
Bulk -0 h
Bulk -3 h
Bulk -6 h
Bulk -12 h
Bulk -18 h
Bulk -24 h
Ex (nm)
Em (nm)

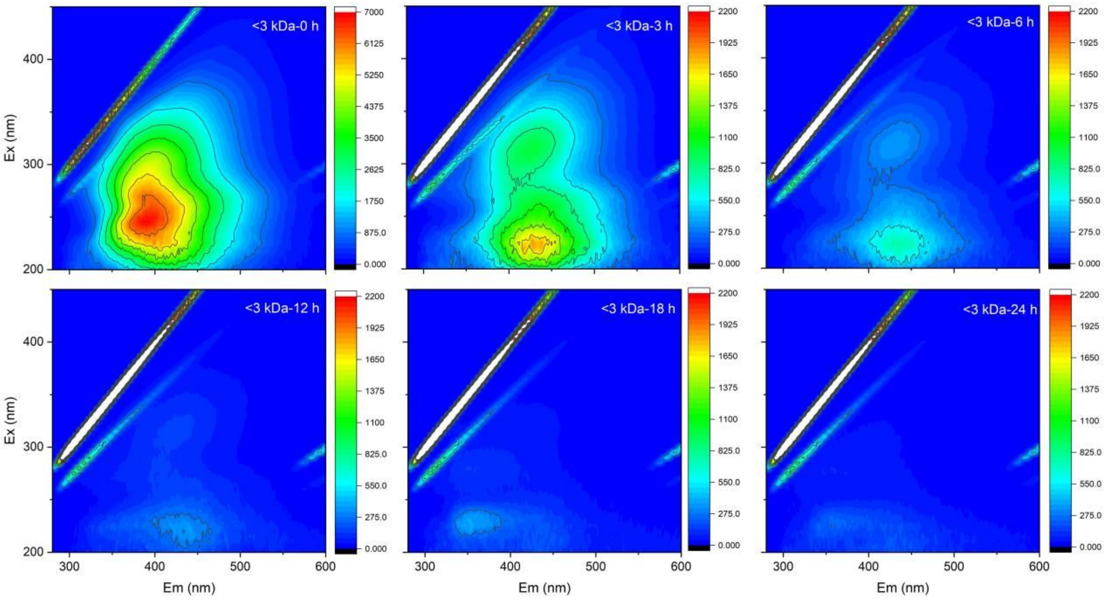
<3 kDa-0 h
<3 kDa-3 h
<3 kDa-6 h
<3 kDa-12 h
<3 kDa-18 h
<3 kDa-24 h
Ex (nm)
Em (nm)

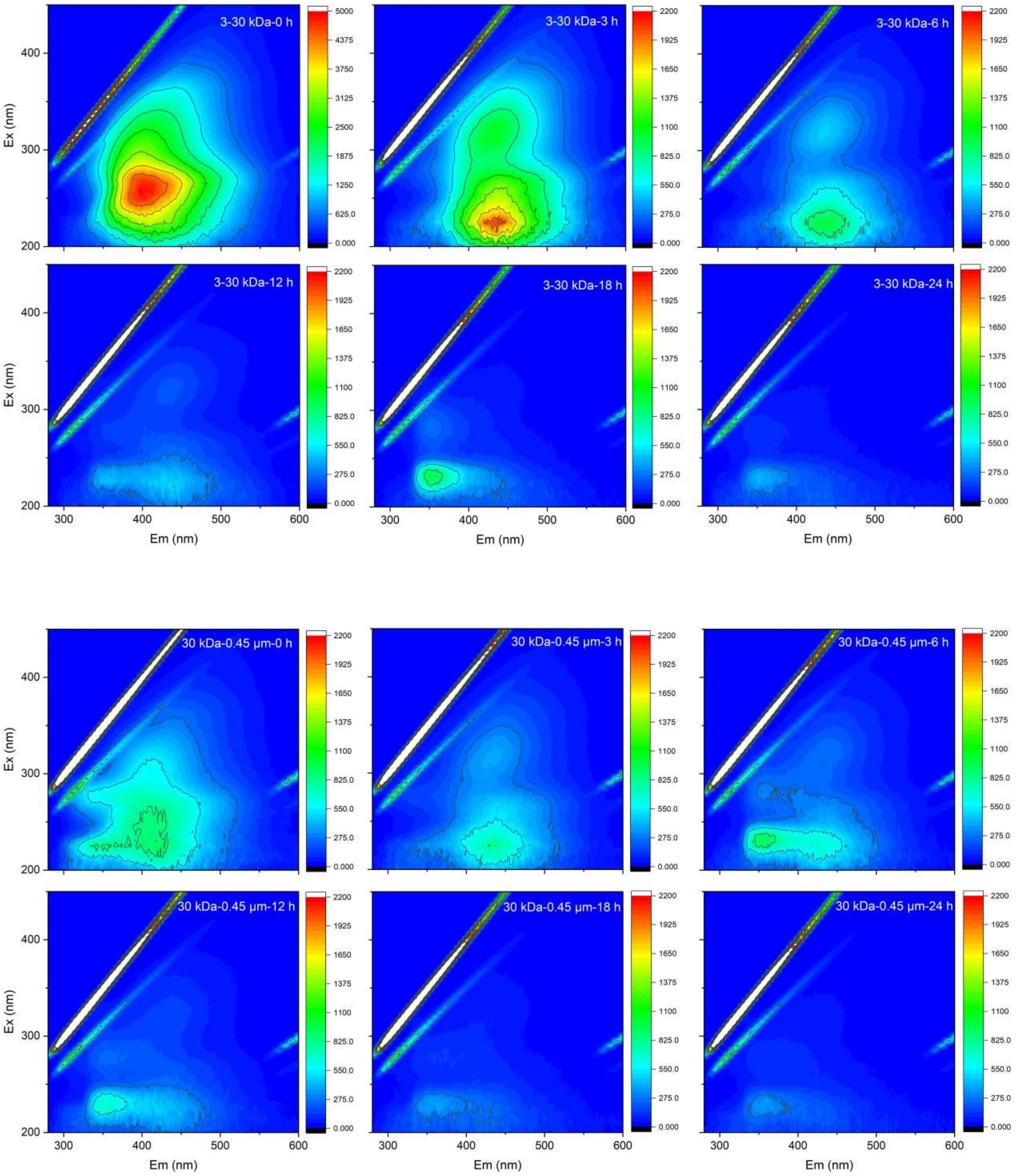

Figure S3. Changes of EEM of bulk DBC and DBC fractions during irradiation time (initial concentration of 5 mg C/L).

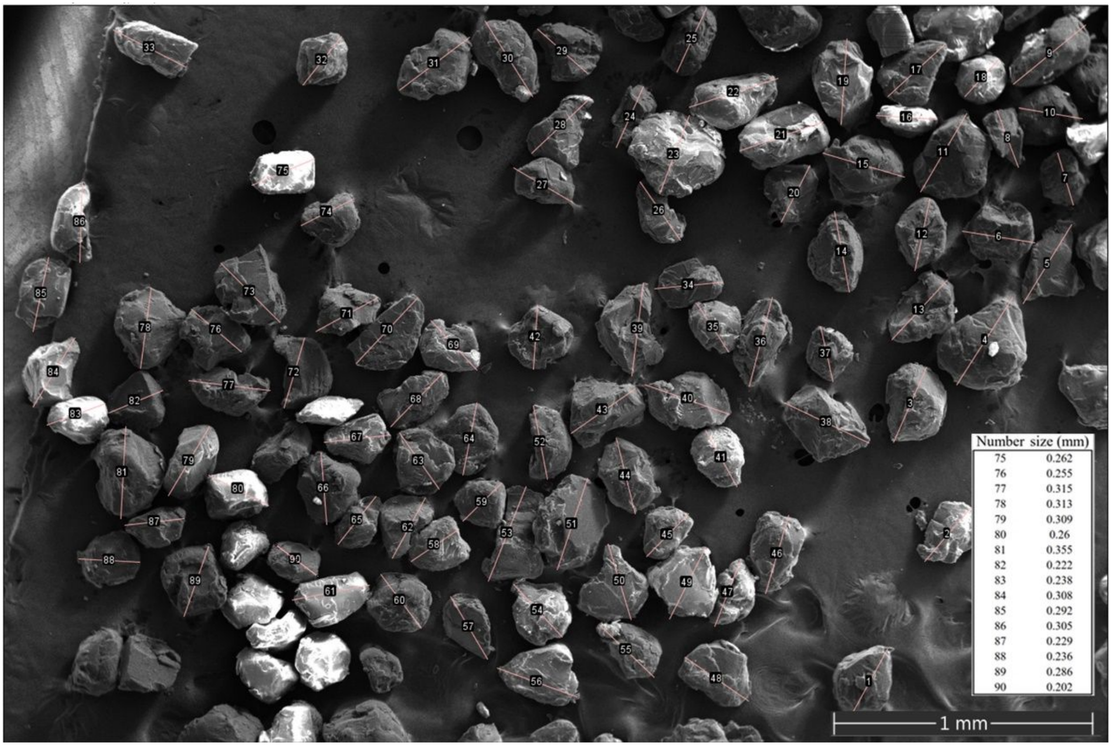

Figure S4. Approaches for measuring PS MPs particle size using ImageJ software (PS MPs aged for 24 h sample was used) [1]

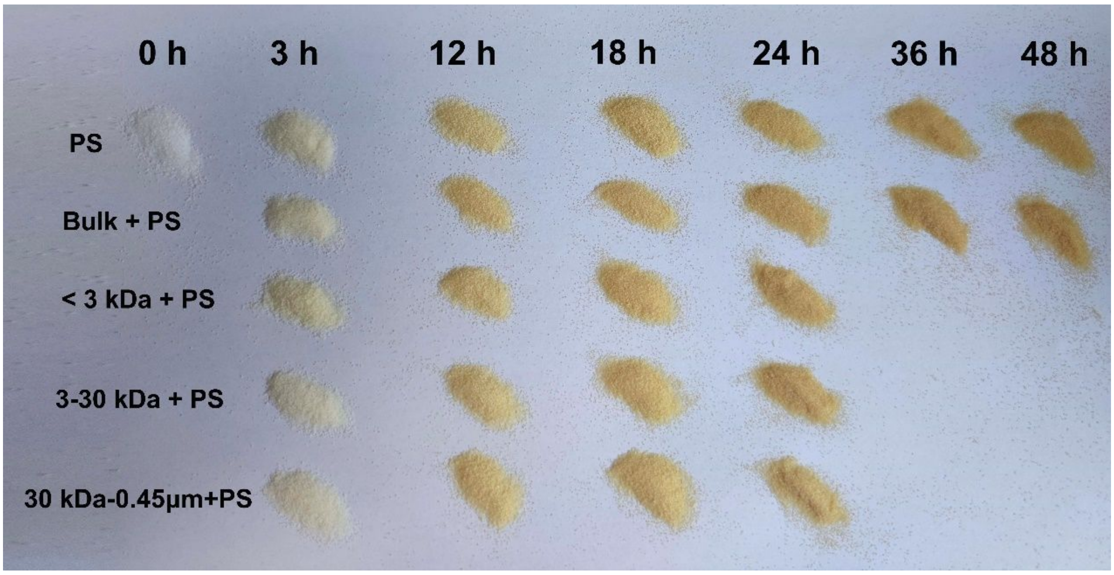

Figure S5. Digital images of PS MPs with or without the mediation of DBC fractions at different exposure times

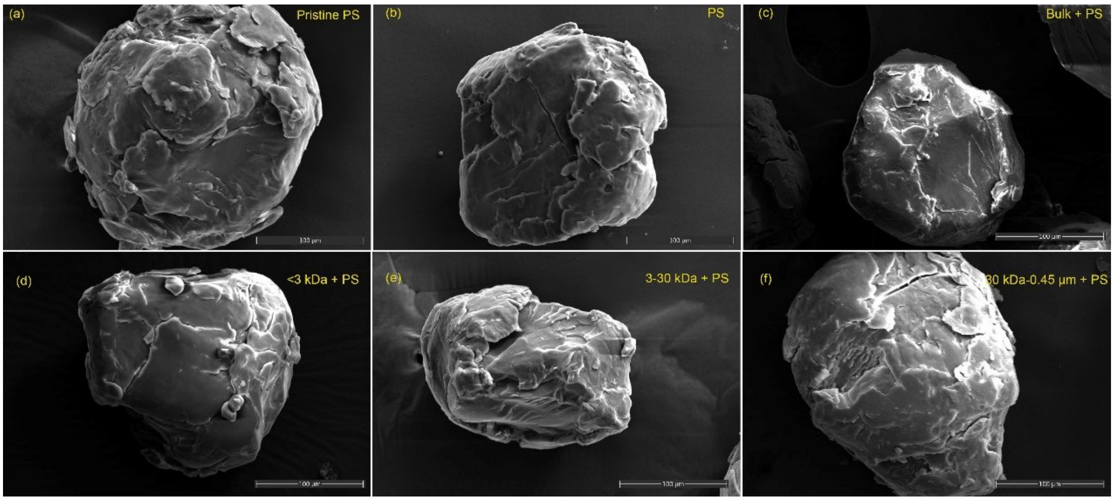

Figure S6. SEM images for PS MPs after 24 h photoaging with or without the presence of DBC

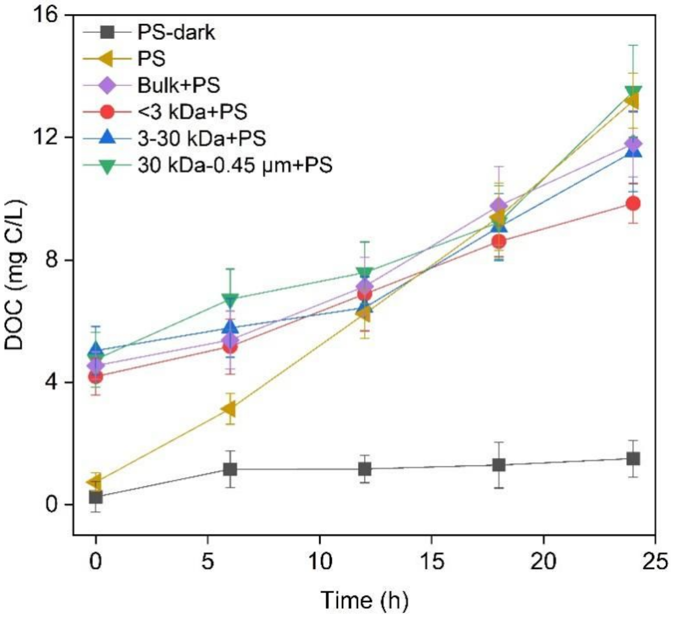

Figure S7. DOC concentration of solution during photoaging

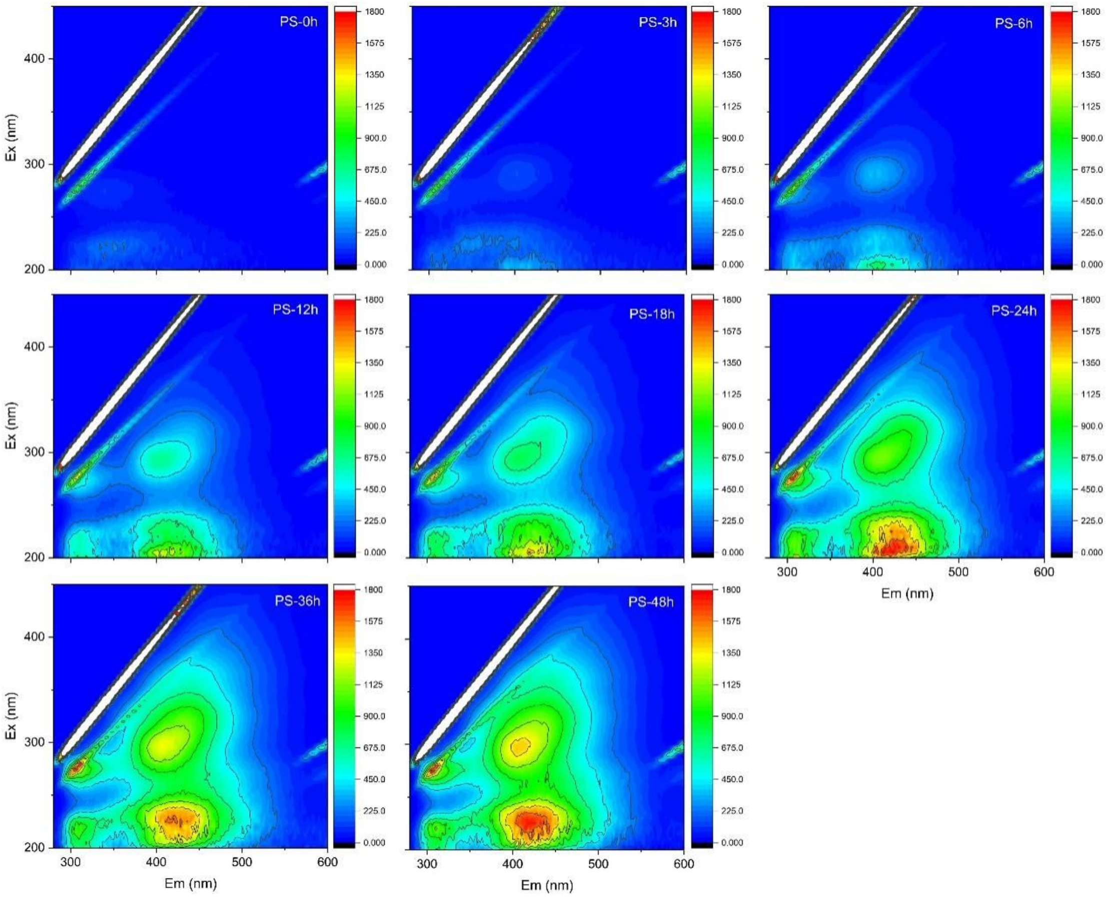

Figure S8. Changes of EEM of PS MPs system during irradiation time

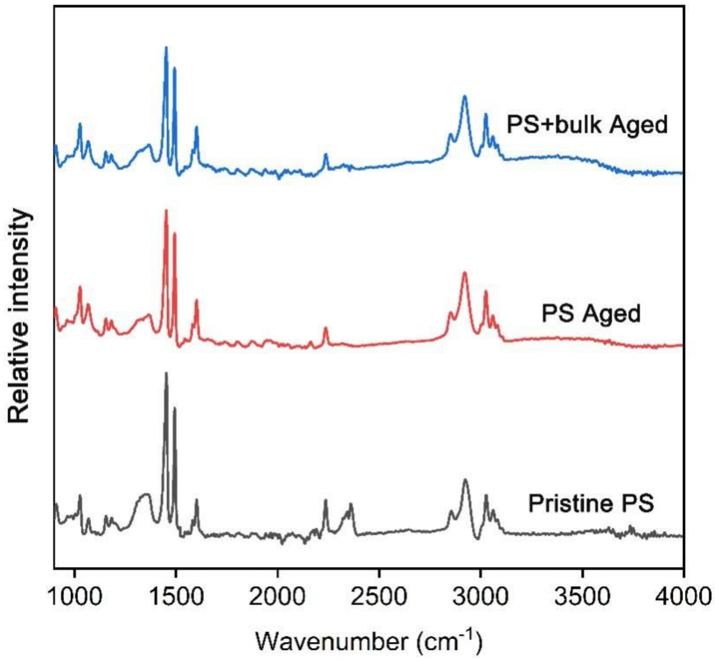

Figure S9. FTIR spectra of PS MPs under natural sunlight exposure with and without bulk DBC

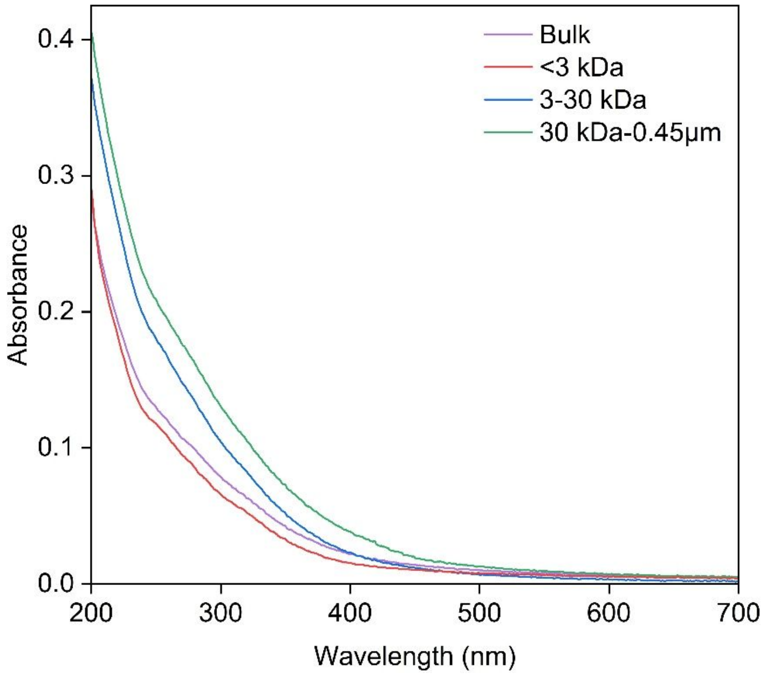

Figure S10. UV-vis absorption spectra of 5 mgC/L DBC and DBC fractions in phosphate buffer

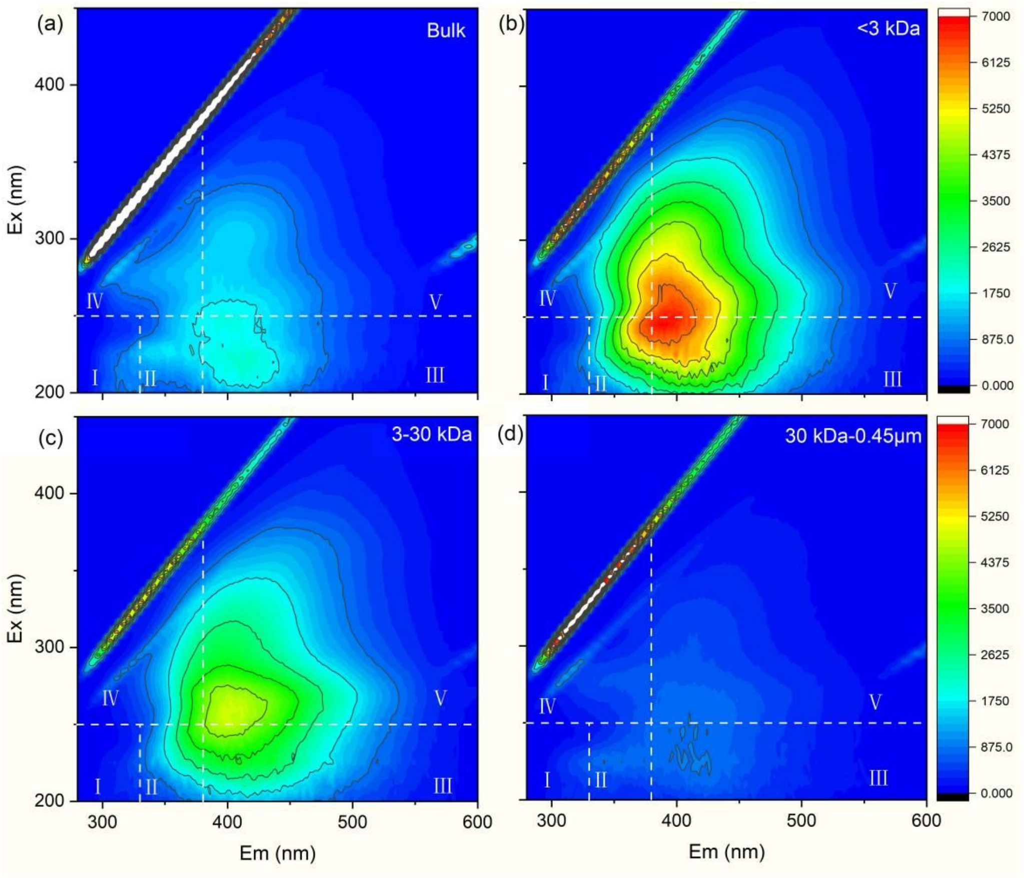

Figure S11. EEM maps of bulk DBC and DBC fractions (5mg C/L)

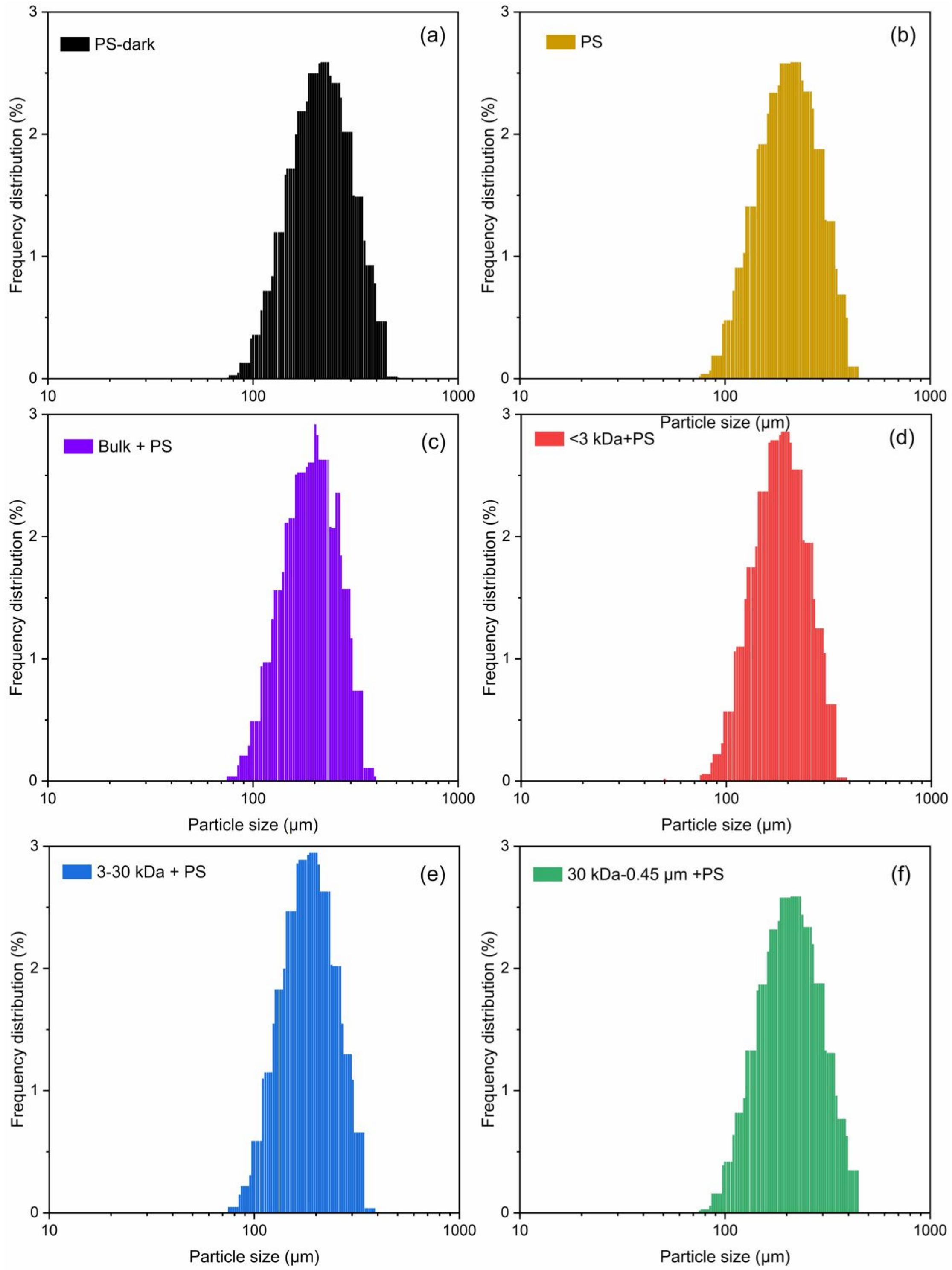

Figure S12. Hydrodynamic size and frequency distributions of PS MPs obtained by LPSA

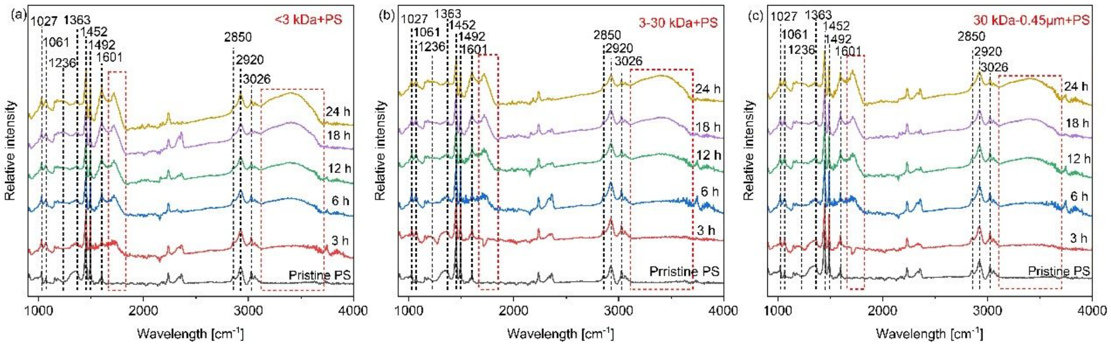

Figure S13. The FTIR spectra of photoaged PS MPs, in the presence of DBC fractions

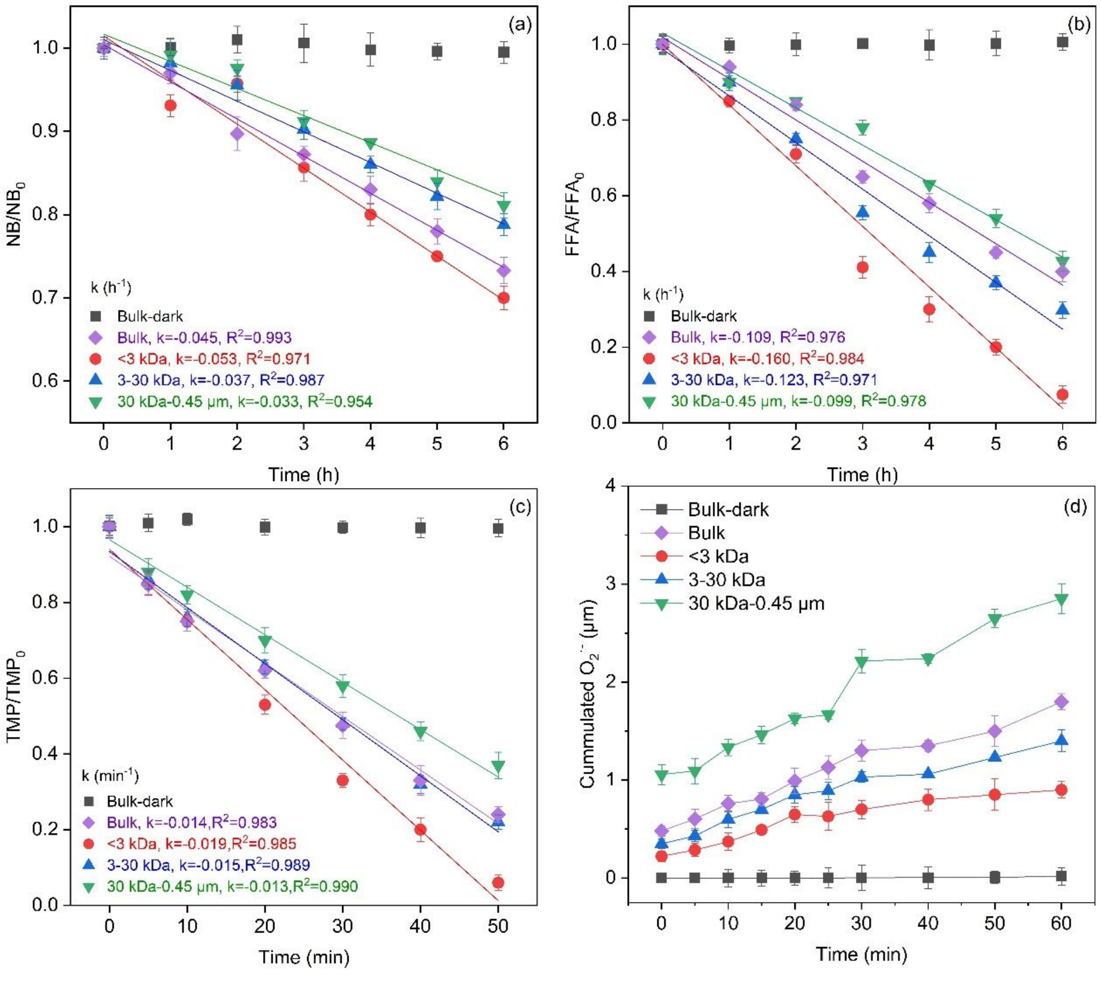

Figure S14. Formation capacity of (a) •OH, (b) $^1O_2$, (c)$^3DBC^*/^3PS^*$ and (d) $O_2•^-$ for bulk DBC and DBC fractions

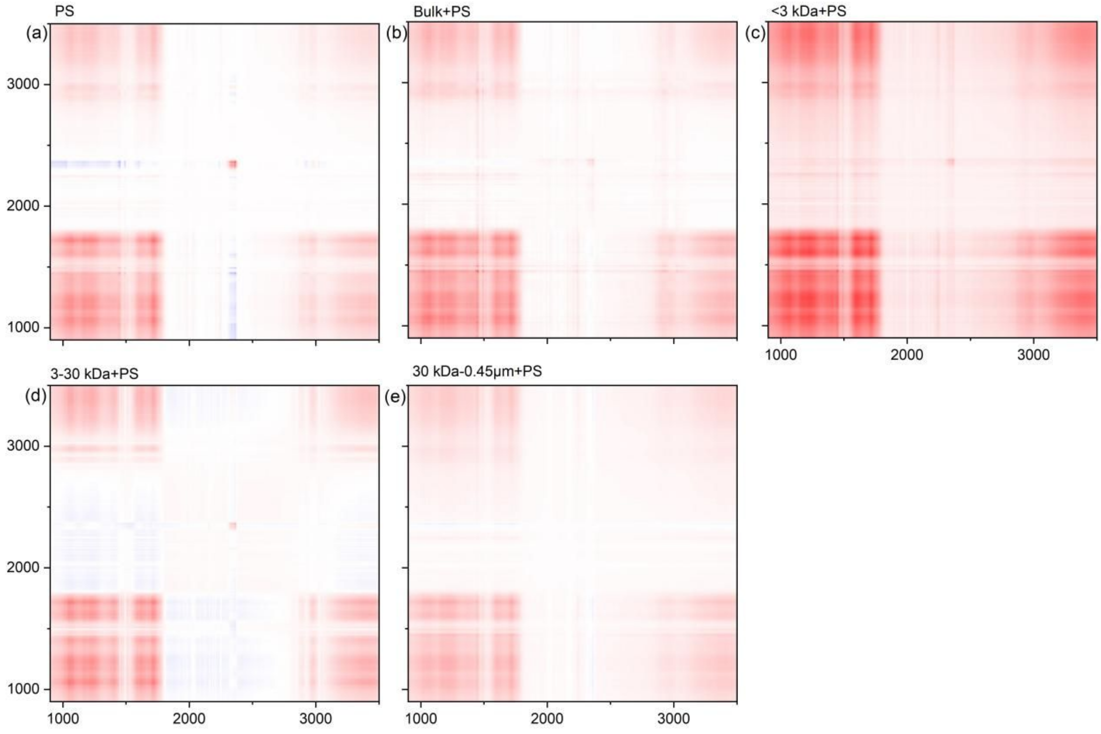

Figure S15. Synchronous 2D-FTIR-COS maps of PS MPs under different incubation times with or without the addition of DBC

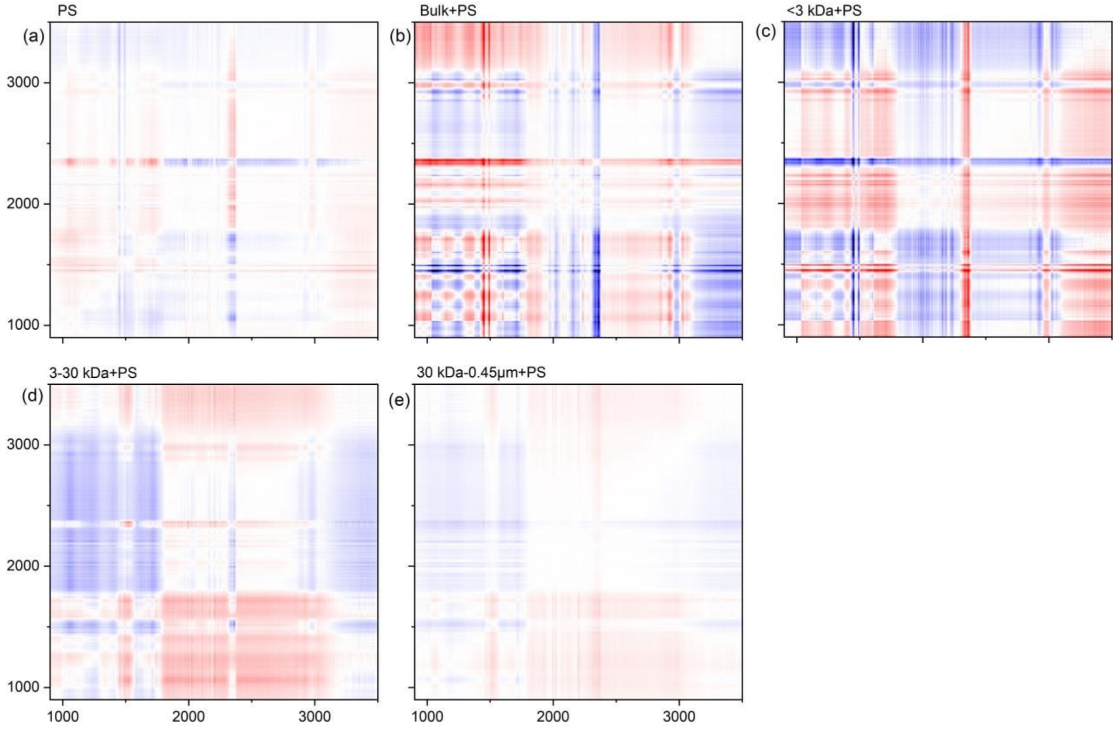

Figure S16. Asynchronous 2D-FTIR-COS maps of PS MPs under different incubation times with or without the addition of DBC

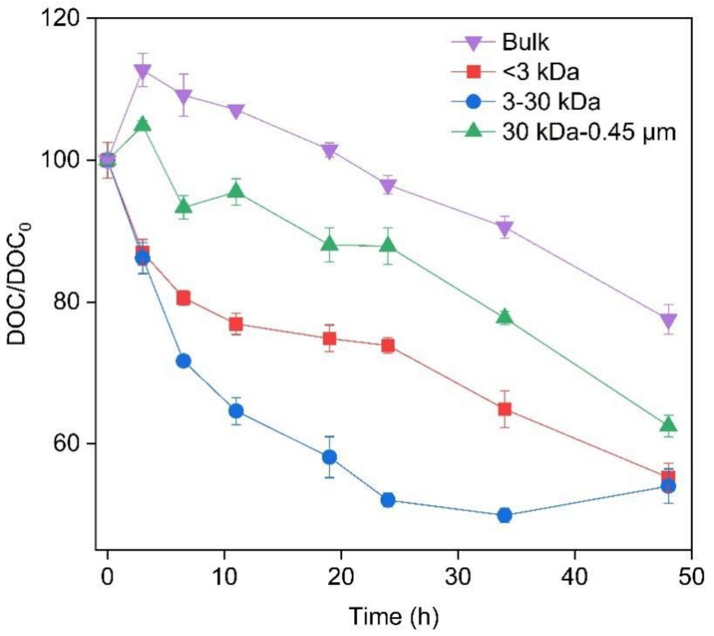

Figure S17. DOC change of bulk DBC or DBC fractions solution

Table S1. Particle size of pristine and photo-aged PS MPs after 48 h aging

	Max	Min	Median	Average	p-value
Pristine PS	448	51	280	281	2.17×10^{-9}
PS	383	42	239	233	-
Bulk + PS	355	26	221	214	0.0014

Note: Data obtained from SEM images, by counting 90 particles using Image J software. The statistical comparison compared to PS group was performed using t-test and $p < 0.05$ was considered significant difference.

Table S1. The characteristics of bulk DBC and DBC fractions

	<3 kDa	3-30 kDa	30 kDa -0.45 μm	Bulk
DOC proportion	48%	30%	18%	-
UV_{254}	0.11	0.18	0.20	0.13
$SUVA_{254}$	0.023	0.035	0.040	0.025
S_{λ}	0.88	0.82	0.80	0.87
E_2/E_3	4.65	4.46	3.55	3.80

Table S3. 2D-COS Data on the assignment and sign of each cross-peak in synchronous (Φ) and asynchronous (Ψ, in the brackets) maps of PS MPs

Peak (cm^{-1})	1363	1452	1492	1601	1717	2850	3026	3393
1363	+	+(+)	+(+)	+(-)	+(-)	+(+)	+(+)	+(+)
1452		+	+(+)	+(-)	+(-)	+(-)	+(+)	+(-)
1492			+	+(-)	+(-)	+(-)	+(+)	+(-)
1601				+	+(-)	+(+)	+(+)	+(+)
1717					+	+(+)	+(+)	+(+)
2850						+	+(+)	+(-)
3026							+	+(-)
3393								+

Table S4. 2D-COS Data on the assignment and sign of each cross-peak in synchronous (Φ) and asynchronous (Ψ, in the brackets) maps of Bulk + PS MPs

Peak (cm^{-1})	1363	1452	1492	1601	1717	2850	3026	3393
1363	+	+(+)	+(+)	+(-)	+(-)	+(+)	+(+)	+(-)
1452		+	+(+)	+(-)	+(-)	+(-)	+(-)	+(-)
1492			+	+(-)	+(-)	+(-)	+(-)	+(-)
1601				+	+(+)	+(+)	+(+)	+(-)
1717					+	+(+)	+(+)	+(-)
2850						+	+(+)	+(-)
3026							+	+(-)
3393								+

Table S5. 2D-COS Data on the assignment and sign of each cross-peak in synchronous (Φ) and asynchronous (Ψ, in the brackets) maps of < 3 kDa + PS MPs

Peak (cm^{-1})	1363	1452	1492	1601	1717	2850	3026	3393
1363	+	+(+)	+(+)	+(-)	+(-)	+(+)	+(+)	+(-)
1452		+	+(+)	+(-)	+(-)	+(-)	+(+)	+(-)
1492			+	+(-)	+(-)	+(-)	+(+)	+(-)
1601				+	+(+)	+(+)	+(+)	+(-)
1717					+	+(+)	+(+)	+(-)
2850						+	+(+)	+(-)
3026							+	+(-)
3393								+

Table S6. 2D-COS Data on the assignment and sign of each cross-peak in synchronous (Φ) and asynchronous (Ψ, in the brackets) maps of 3-30 kDa + PS MPs

Peak (cm^{-1})	1363	1452	1492	1601	1717	2850	3026	3393
1363	+	+(+)	+(+)	+(-)	+(-)	+(+)	+(+)	+(-)
1452		+	+(+)	+(-)	+(-)	+(-)	+(-)	+(-)
1492			+	+(-)	+(-)	+(-)	+(-)	+(-)
1601				+	+(+)	+(+)	+(+)	+(-)
1717					+	+(+)	+(+)	+(-)
2850						+	+(+)	+(-)
3026							+	+(-)
3393								+

Table S7. 2D-COS Data on the assignment and sign of each cross-peak in synchronous (Φ) and asynchronous (Ψ, in the brackets) maps of 30 kDa-0.45 μm + PS MPs

Peak (cm^{-1})	1363	1452	1492	1601	1717	2850	3026	3393
1363	+	+(+)	+(+)	+(-)	+(-)	+(+)	+(+)	+(-)
1452		+	+(+)	+(-)	+(-)	+(-)	+(-)	+(-)
1492			+	+(-)	+(-)	+(-)	+(-)	+(-)
1601				+	+(+)	+(+)	+(+)	+(-)
1717					+	+(+)	+(+)	+(-)
2850						+	+(+)	+(-)
3026							+	+(-)
3393								+

References

1. Wu, X.; Liu, P.; Gong, Z.; Wang, H.; Huang, H.; Shi, Y.; Zhao, X.; Gao, S., Humic Acid and Fulvic Acid Hinder Long-Term Weathering of Microplastics in Lake Water. *Environ Sci Technol* **2021,** *55*, (23), 15810-15820.
2. Zhu, K.; Jia, H.; Sun, Y.; Dai, Y.; Zhang, C.; Guo, X.; Wang, T.; Zhu, L., Long-term phototransformation of microplastics under simulated sunlight irradiation in aquatic environments: Roles of reactive oxygen species. *Water Res* **2020,** *173*, 115564.
3. Zhou, Z.; Chen, B.; Qu, X.; Fu, H.; Zhu, D., Dissolved Black Carbon as an Efficient Sensitizer in the Photochemical Transformation of 17beta-Estradiol in Aqueous Solution. *Environ Sci Technol* **2018,** *52*, (18), 10391-10399.
4. Chen, C.-Y.; Jafvert, C. T., Photoreactivity of carboxylated single-walled carbon nanotubes in sunlight: reactive oxygen species production in water. *Environmental science technology* **2010,** *44*, (17), 6674-6679.
5. Haag, W. R. H., Juerg, Singlet oxygen in surface waters. 3. Photochemical formation and steady-state concentrations in various types of waters. *Environmental science technology* **1986,** *20*, (4), 341-348.
6. Hua, Z.; Guo, K.; Kong, X.; Lin, S.; Wu, Z.; Wang, L.; Huang, H.; Fang, J., PPCP degradation and DBP formation in the solar/free chlorine system: Effects of pH and dissolved oxygen. *Water Research* **2019,** *150*, 77-85.
7. Fang, J.; Fu, Y.; Shang, C., The Roles of Reactive Species in Micropollutant Degradation in the UV/Free Chlorine System. *Environmental Science & Technology* **2014,** *48*, (3), 1859-1868.
8. Sutherland, M. W.; Learmonth, B. A., The tetrazolium dyes MTS and XTT provide new quantitative assays for superoxide and superoxide dismutase. *Free radical research* **1997,** *27*, (3), 283-289.
9. Stark, N. M.; Matuana, L. M., Surface chemistry changes of weathered HDPE/wood-flour composites studied by XPS and FTIR spectroscopy. *Polymer Degradation and Stability* **2004,** *86*, (1), 1-9.
10. Devi, R. R.; Maji, T. K., Effect of Nano-ZnO on Thermal, Mechanical, UV Stability, and Other Physical Properties of Wood Polymer Composites. *Industrial & Engineering Chemistry Research* **2012,** *51*, (10), 3870-3880.
11. Bayo, J.; Rojo, D.; Olmos, S., Weathering indices of microplastics along marine and coastal sediments from the harbor of Cartagena (Spain) and its adjoining urban beach. *Mar Pollut Bull* **2022,** *178*, 113647.
12. Hofko, B.; Porot, L.; Falchetto Cannone, A.; Poulikakos, L.; Huber, L.; Lu, X.; Mollenhauer, K.; Grothe, H., FTIR spectral analysis of bituminous binders: reproducibility and impact of ageing temperature. *Materials and Structures* **2018,** *51*, (2), 45.
13. Chercoles Asensio, R.; San Andres Moya, M.; de la Roja, J. M.; Gomez, M., Analytical characterization of polymers used in conservation and restoration by ATR-FTIR spectroscopy. *Anal Bioanal Chem* **2009,** *395*, (7), 2081-96.
14. Olmos, D.; Martin, E. V.; Gonzalez-Benito, J., New molecular-scale information on polystyrene dynamics in PS and PS-BaTiO3 composites from FTIR spectroscopy. *Phys Chem Chem Phys* **2014,** *16*, (44), 24339-49.
15. Liu, S.; Cui, Z.; Ding, D.; Bai, Y.; Chen, J.; Cui, H.; Su, R.; Qu, K., Effect of the molecular weight of DOM on the indirect photodegradation of fluoroquinolone antibiotics. *J Environ Manage* **2023,** *348*, 119192.

16. Sun, Q.; Wang, G.; Yin, R.; Zhang, T.; Zheng, Y.; Wu, C.; Liu, C.; Huang, K.; Wang, F., Effects of molecular-level component variation of fulvic acid on photodegradation of Microcystin-LR under solar irradiation. *Chemical Engineering Journal* **2022,** *449*, 137553.
